# Untargeted metabolomic and lipidomic analyses reveal lipid dysregulation in the plasma of acute leukemia patients

**DOI:** 10.3389/fmolb.2023.1235160

**Published:** 2023-11-10

**Authors:** Cindy Arévalo, Laura Rojas, Mary Santamaria, Luisana Molina, Lina Arbeláez, Paula Sánchez, Ricardo Ballesteros-Ramírez, Monica Arevalo-Zambrano, Sandra Quijano, Mónica P. Cala, Susana Fiorentino

**Affiliations:** ^1^ Grupo de Inmunobiología y Biología Celular, Facultad de Ciencias, Pontificia Universidad Javeriana, Bogotá, Colombia; ^2^ MetCore—Metabolomics Core Facility, Vice-Presidency for Research, Universidad de Los Andes, Bogotá, Colombia; ^3^ Hospital Universitario San Ignacio, Bogotá, Colombia

**Keywords:** untargeted metabolomics, acute leukemia, lipids, antioxidant capacity, acute myeloid leukemia, acute lymphoid leukemia

## Abstract

Acute leukemias (AL) are aggressive neoplasms with high mortality rates. Metabolomics and oxidative status have emerged as important tools to identify new biomarkers with clinical utility. To identify the metabolic differences between healthy individuals (HI) and patients with AL, a multiplatform untargeted metabolomic and lipidomic approach was conducted using liquid and gas chromatography coupled with quadrupole-time-of-flight mass spectrometry (LC-QTOF-MS or GC-QTOF-MS). Additionally, the total antioxidant capacity (TAC) was measured. A total of 20 peripheral blood plasma samples were obtained from patients with AL and 18 samples from HI. Our analysis revealed 135 differentially altered metabolites in the patients belonging to 12 chemical classes; likewise, the metabolic pathways of glycerolipids and sphingolipids were the most affected in the patients. A decrease in the TAC of the patients with respect to the HI was evident. This study conducted with a cohort of Colombian patients is consistent with observations from other research studies that suggest dysregulation of lipid compounds. Furthermore, metabolic differences between patients and HI appear to be independent of lifestyle, race, or geographic location, providing valuable information for future advancements in understanding the disease and developing more global therapies.

## Introduction

AL are a heterogeneous group of hematological malignancies that involve blocked hematopoietic progenitors and accumulate in the early phases of cell differentiation, leading to marrow failure, and can be classified into two large groups: acute myeloid leukemia (AML) and acute lymphoid leukemia (ALL) (B-ALL or T-ALL) (Arber et al., 2016). In the process of clonal evolution, leukemic cells accumulate mutations that can lead to aberrant metabolic programs that are necessary to meet bioenergetic and biosynthetic demands and maintain a redox balance for tumor survival and proliferation (DeBerardinis and Chandel, 2016; Romer-Seibert and Meyer, 2021). In the Colombian population, remission rates are low compared to those in developed countries, so exploring the metabolic profiles in these cases is intriguing (Combariza et al., 2007; Ballesteros-Ramírez et al., 2020; Sossa et al., 2021). Increased glucose consumption by leukemic progenitor cells is beneficial for producing metabolic intermediates involved in other metabolic pathways and for ATP generation. On the other hand, leukemia-initiating cells (LICs) in AML are characterized by their dependence on oxidative phosphorylation and branched-chain amino acids, low glucose consumption, low production of reactive oxygen species (ROS), and high levels of glutathione (GSH) (Jones et al., 2018). While in B-ALL, LICs that carry alterations in the PAX5 (Paired Box 5) or IKZF (IKAROS Family Zinc Finger 1) genes are related to unlimited glucose consumption (Boag et al., 2006).

The evaluation of specialized tumor metabolism can be measured at the systemic level by evaluating the TAC and using metabolomic platforms, such as mass spectrometry (MS) and nuclear magnetic resonance (NMR), to identify unique metabolic fingerprints, which may allow the identification of biomarkers and therapeutic targets (Muthu and Nordström, 2019; Schmidt et al., 2021). Some authors have demonstrated its usefulness in blood plasma for diagnosis (Morad et al., 2022), response to treatment and follow-up of patients with AL (Naz et al., 2013; Grønningsæter et al., 2019; Kim et al., 2021), and to assess cellular response to antileukemic agents (Dhakshinamoorthy et al., 2015). In this sense, metabolic differences between HI and adult patients with AL have been reported using ^1^H NMR (Musharraf et al., 2016; Yang et al., 2021). Other authors have managed to define groups of metabolites (mainly energy) associated with prognosis in patients with AML using gas chromatography-time-of-flight mass spectrometry (GC-TOF-MS) or liquid chromatography-mass spectrometry (LC-MS) (Chen et al., 2014; Dong et al., 2019). However, there are few publications describing differential metabolites between ALL and AML by LC-MS or ^1^H NMR (Musharraf et al., 2017; Hao et al., 2022). In addition, few studies have focused on the oxidative stress profile in AL. Particularly, Naz et al. (2013) showed that patients in complete remission decreased their total antioxidant status (Naz et al., 2013); however, in pediatric patients with ALL, the differences in the levels of some antioxidants are not clear (Olaniyi et al., 2011).

Tumor metabolism is influenced by intrinsic factors, such as genetic alterations or lineage/tissue of origin, and extrinsic factors, such as access to nutrients and oxygen, interaction with cells in the microenvironment, and exposure to radiation or chemotherapy (Vander Heiden and DeBerardinis, 2017). In other words, population characteristics such as race, genetics, food culture, or healthy habits can also influence tumor metabolism and have great relevance to the risk of development or progression of the disease, the risk of recurrence, the risk of disease, and mortality for some types of cancer (Faulds and Dahlman-Wright, 2012; Islami et al., 2018; Peng et al., 2022).

Based on the aforesaid considerations, the current study examined the metabolomic profiles of cohorts comprising Colombian patients diagnosed with AL, using an untargeted metabolomic and lipidomic approach by LC-QTOF-MS and GC-QTOF-MS. Furthermore, the study established the total antioxidant capacities within these cohorts. To our knowledge, this is the first report to investigate the metabolic and lipid alterations associated with AL, specifically in the Colombian population. These findings hold significant potential for the development of future diagnostic and prognostic biomarkers for this population.

## Materials and methods

### Study participants

Between 2019 and 2020, twenty patients with AL who attended the Hospital Universitario San Ignacio (Bogotá D.C., Colombia) were linked to this study. They were patients older than 18 years, who were diagnosed for the first time and had not received previous therapy. The study was approved by the Ethics Committee of the Hospital Universitario San Ignacio and the Centro Javeriano de Oncología (Bogotá D.C., Colombia). Following the Declaration of Helsinki, written informed consent was obtained from all participants prior to clinical data collection and sample collection. The diagnosis was made according to the World Health Organization classification of tumors of hematopoietic and lymphoid tissues 2017 (Arber et al., 2016).

### Sample collection

Peripheral blood samples were obtained from twenty patients with a *de novo* diagnosis of acute leukemia before starting chemotherapy treatment. Samples were collected with a minimum fast of 8 h in K_2_EDTA tubes, and peripheral blood was centrifuged exactly 4 h after collection, at 3,500 rpm at 4°C for 10 min. The plasma obtained was aliquoted and stored at −80°C until processing. As a control group, 18 plasmas were collected from HI matched by age and sex under the same conditions. According to the NCCN Clinical Practice Guidelines in Oncology ^®^ (Chang et al., 2021; Pollyea et al., 2021) and given the number of samples collected, the response to treatment at the end of induction was divided into two groups of patients: Complete remission (CR) [including CR with negative EMR (minimal residual disease)] and non-responders (NR) (including CR with a partial hematological response (CRp), CR with an incomplete hematological response (Cri) and CR with positive EMR or unknown), patients with premature death and NR).

### Untargeted metabolomic and lipidomic analysis

#### Metabolomic analysis by LC-QTOF-MS and GC-QTOF-MS

For metabolomic analysis by reverse phase liquid chromatography coupled to mass spectrometry with a time-of-flight analyzer (RP-LC-QTOF-MS), samples were extracted using 40 µL of plasma mixed with 120 µL of cold methanol and ethanol (1:1, v/v) (−20°C) and samples were vortex-mixed for 5 min. Samples were incubated for 20 min at −20°C to precipitate proteins. Then, the samples were centrifuged for 10 min (16,000 g, 4°C). Metabolomic analysis was performed using the UHPLC system (Agilent 1260 Infinity LC System) coupled with the Q-TOF LC/MS system (Agilent Technologies, Waldronn, Germany) equipped with an electrospray ionization (ESI) source 2 µL of the extracted sample was injected into the InfinityLab Poroshell 120 EC-C18 (100 mm × 3.0 mm, 2.7 µm) column at 30°C using 0.1% (v/v) formic acid in water (A) and 0.1% (v/v) formic acid in acetonitrile (B) as a mobile phase with a flow rate of 0.3 mL/min. Gradient elution started with 25% B and increased to 95% within 35 min. Then, the gradient returned to initial conditions at 35.1 min and held there for 8 min to allow column re-equilibrium. For constant mass correction, two reference masses were used and continuously infused into the system: *m/z* 121.0509 (C_5_H_4_N_4_ + H)^+^ and m/z 922.0098 (C_18_H_18_O_6_N_3_P_3_F_24_ + H)^+^ for positive ionization mode (ESI+) and *m/z* 112.9856 (C_2_O_2_F_3_ – NH_4_)^-^ and *m/z* 1033.9881 (C_18_H_18_O_6_N_3_P_3_F_24_ + FA-H)^-^ for negative ionization mode (ESI -). The system was operated in full scan mode from 100 to 1,100 *m/z*; the capillary voltage was set to 3000, the drying gas flow rate was 12 L/min at 290°C, the gas nebulizer 52 psi, fragmentor voltage was 175 V and the skimmer 65 V and octopole radio frequency voltage (OCT RF Vpp) 750 V for both, positive and negative ionization modes. Data were collected in centroid mode at a scan rate of 1.02 spectrum per second.

For metabolomic analysis by gas chromatograph coupled to mass spectrometry with a time-of-flight analyzer (GC-QTOF-MS), samples were extracted using 140 µL of plasma mixed with 420 µL of cold methanol (−20°C) and vortex-mixed for 5 min. Samples were incubated for 20 min at −20°C to precipitate proteins. Then, the samples were centrifuged for 10 min (16,000 g, 4°C). An aliquot of 100 µL was transferred into glass inserts and evaporated to dryness in a speed vacuum concentrator (Thermo Scientific). The dry residue was dissolved in 10 µL of methoxyamine hydrochloride in pyridine (15 mg/mL) and vortex-mixed for 5 min. The samples were incubated for 16 h at room temperature in the dark. The silylation process was followed by adding 10 µL of bis(trimethylsilyl)trifluoroacetamide (BSTFA) with 1% trimethylchlorosilane (TMCS). After vortex-mixing (5 min) and incubation for 1 h at 70°C, the samples were diluted with 50 µL of internal standard (methyl stearate in heptane C18:0, 10 ppm). GC-QTOF-MS experiments were performed on an Agilent Technologies 7890B GC system coupled to 7250 QTOF mass spectrometer system (Agilent Technologies). Derivatized samples were injected (1 µL) with a split ratio of 30:1 onto an HP-5MS capillary column (30 m × 0.25 mm; 0.25 µm) (Agilent Technologies) at a constant gas flow (helium) of 0.7 mL/min. The injector temperature was 250°C. The temperature gradient was kept at 60°C for 1 min and then programmed to 320°C at 10°C/min. Mass spectra were recorded at 70 eV in full scan mode with *m/z* values ranging from 50 to 600. The transfer line, filament source, and quadrupole temperature were fixed at 280°C, 230°C, and 150°C, respectively.

#### Lipidomic analysis by LC-QTOF-MS

For lipids extraction, 100 µL of plasma was extracted with 350 µL of cold methanol and 350 µL of MTBE and vortex mixed for 5 min. Then, the samples were centrifuged at 13,000 g for 10 min at room temperature. Lipidomic analysis was performed using the same RP-LC–QTOF–MS system employed for metabolomics analysis. A 1 µL of the extracted sample was injected into the InfinityLab Poroshell 120 EC-C8 (100 mm × 2.1 mm, 2.7 µm) column at 60°C using 5 mM ammonium formate in Mili-Q water) (A) and 5 mM ammonium formate in isopropanol: methanol (15:85) (B) as a mobile phase with a 0.4 mL/min flow rate. Gradient elution started with 75% B, then increased to 96% within 23 min, and kept there for 13 min, then increased to 100% and kept constant for 4 min. Then, the gradient returned to initial conditions at 42 min and held there for 11 min to allow column re-equilibrium. The same reference masses were used throughout the analysis as described in metabolomics analysis by LC-QTOF-MS for positive and negative ionization modes. The system was operated in full scan mode from 100 to 1,800 *m/z*; a capillary voltage was set to 3,000 V, the drying gas flow rate was 12 L/min at 290°C; and the gas nebulizer 45 psi, fragmentor voltage 175 V, the skimmer 65 V and octopole radio frequency voltage (OCT RF Vpp) 750 V. Data were collected in centroid mode at a scan rate of 1.02 spectrum per second.

#### Quality assurance (QA) and quality control (QC) procedures

Quality assurance and quality control procedures were implemented according to published guidelines to reduce unwanted variation (26) (Kirwan et al., 2022). Pure solvents and extraction blanks were evaluated at the beginning of each sequence to ensure the cleanliness of equipment and materials used in sample preparation. To equilibrate the chromatographic system, pooled samples (QC) were injected, which were prepared by mixing equal volumes of each plasma sample using the same procedure for both metabolomic (LC and GC) and lipidomic analysis. To monitor the system’s stability, these QC samples were injected every ten samples. Additionally, biological samples were randomized within the sequence to reduce the possibility of bias (Sumner et al., 2007).

#### Data treatment

All raw LC-QTOF-MS datasets were processed using *Agilent MassHunter Profinder B.10.0* Software for deconvolution, alignment, and integration, using algorithms such as molecular feature extraction and recursive feature extraction; then, the raw data were inspected manually to remove background noise and unrelated ions. For GC-QTOF-MS, samples were normalized by internal standards prior to the statistical analysis. Finally, for all platforms, the data was filtered by presence and reproducibility, and the coefficient of variation (CV) in the QC lower than 20% to LC (or 30% to GC) was used for statistical analysis.

#### Statistical analysis

For both LC-MS and GC-MS data, the identification of the molecular characteristics with statistical differences between HI and AL patients were carried out using univariate and multivariate statistical analysis. First, the *p-*value was determined by Mann-Whitney U test (nonparametric tests) with a Benjamini-Hochberg False Discovery Rate *post hoc* correction (FDR) using MatLab (R2019b, Mathworks, Inc., Natick), while for the multivariate analysis, an unsupervised principal component analysis (PCA) and orthogonal partial least squares regression (OPLS-DA) was applied using SIMCA-P + 16.0 software. The statistically significant variables were selected based on *p*-value with FDR *p* < 0.05 and variance important in projection (VIP) > 1 with Jack-knife confident interval (JK).

#### Metabolites identification

The identification of metabolites was carried out based on a 4-level confidence system for high-resolution mass spectrometry analysis following the parameters (Schymanski et al., 2014). Metabolites by LC-MS were annotated using various online database (http://hmdb.ca), (http://genome.jp/keg), (https://massbank.eu/MassBank/), (http://lipidmaps.org) and (http://metlin.scripps.edu) utilized for this purpose CEU Mass Mediator tool (http://ceumass.eps.uspceu.es/). The metabolite’s identity was confirmed by iterative MS/MS data with Agilent Lipid Annotator software, MS-DIAL 4.80 (http://prime.psc.riken.jp/compms/msdial/main.html), and CFM-ID 4.0 (https://cfmid.wishartlab.com/) for *in silico* mass spectral fragmentation. For GC-QTOF-MS chromatograms were deconvoluted and compared with Fiehn GC-MS Metabolomics RTL Library (Kind et al., 2009).

#### Pathway analysis

Metabolic pathway analysis was performed with the MetaboAnalyst 5.0 tool (http://www.MetaboAnalyst.ca/), integrating enrichment and topology pathway approaches. A list of identified significant metabolite compound names was loaded and processed using the “*Homo sapiens*” library. The KEGG pathway information was obtained in October 2019, and the specific pathway analysis parameters were the visualization method by scatter plot (testing significant features), enrichment method (hypergeometric test), topology analysis (relative-betweenness centrality), and selecting a pathway library by *H. sapiens*.

### Determination of TAC in plasma

The antioxidant capacity was evaluated using the e-BQClab device (Bioquochem, Asturias, Spain) that measures the redox potential, which is expressed in micro coulombs (μC). The results in μC were transformed to Trolox Equivalent Antioxidant Capacity Units (TEAC). The e-BQClab device using electrochemistry can distinguish between fast and slow antioxidants: the Q1 value refers to the antioxidant capacity of the compounds with the highest free radical scavenging rate (examples, uric acid, GSH, vitamin E), while the Q2 value refers to the antioxidant capacity of the compounds with the lowest rate of free radical uptake (examples, polyphenols, resveratrol). The QT value is the sum of both. The measurement was performed in duplicate using 50 μL of all collected plasma samples.

## Results

### Characteristics of AL patients and HI

The mean age at diagnosis of the patients evaluated was 45.8 years (range 21–76), and 55% were female patients. We collected 9 patients with B-ALL, 9 patients with AML, 1 patient with acute promyelocytic leukemia, and 1 patient with mixed-phenotype acute leukemia (B-lymphoid and myeloid differentiation). The karyotype was normal in 40% (8) of the patients, abnormal in 50% (10), and there was no growth in 10% (2). According to the risk categories, 80% of the patients were classified as high-risk and the remaining 20% as intermediate-risk. The mean white blood cell count was 76,786 cells/µL (interval 800–403,000), hemoglobin was 9.0 g/dL (3.2–12), platelets were 68,130 cells/µL (interval 8,300–228,000), and the mean number of tumor cells over the total nucleated cells in bone marrow was 68.8% (interval 20–93.9). The first phase of chemotherapy (induction therapy) was based on the PETHEMA protocols for all patients, 7x3 or 5-Azacytidine for AML patients, and AIDA-PETHEMA for acute promyelocytic leukemia patients. Of the total number of patients at the end of induction, 10% (2) achieved CR, 70% (14) were NR, and 20% (4) could not be evaluated. In total, 18 samples were collected from HI; the group had a mean age of 31.9 years (range 19–61), and 55% were women. The clinical and demographic data of the patients are summarized in Table 1, along with the HI data. Clinical data related to treatment and response are detailed in Supplementary Table S1.

**TABLE 1:** Summary description of the clinical characteristic of AL patients and date of healthy individuals.

Characteristic	*n* (%)
Sex (female)	11 (55)
Age (years), median (interval)	45.8 (21–76)
Immunophenotype	
B-ALL	9 (45)
AML	9 (45)
B/M AL	1 (5)
AML M3	1 (5)
Karyotype	
Normal	8 (40)
Abnormal	10 (50)
No growth	2 (10)
Risk	
Intermedium	4 (20)
High	16 (80)
Hematological parameters	
WBC count (µL), median (interval)	76,786 (800–403,000)
Hb (g/dL), median (interval)	9.0 (3.2–12)
Platelet count (µL), median (interval)	68,130 (8,300–228,000)
Tumor cells in bone marrow (%), median (interval)	68,8 (20–93,9)
Healthy Individuals	
Sex (female)	10 (55)
Age (years), median (interval)	31.9 (19–61)

B-ALL, B-acute lymphoid leukemia; AML, Acute myeloid leukemia; B/M AL, mixed-phenotype acute leukemia patient (B-lymphoid and myeloid differentiation); AML M3, Promyelocytic—acute myeloid leukemia; Hb, Hemoglobin; WBC, White blood cells.

### Alterations in lipid metabolism at the plasma level, differentiate HI from AL patients

**FIGURE 1:**
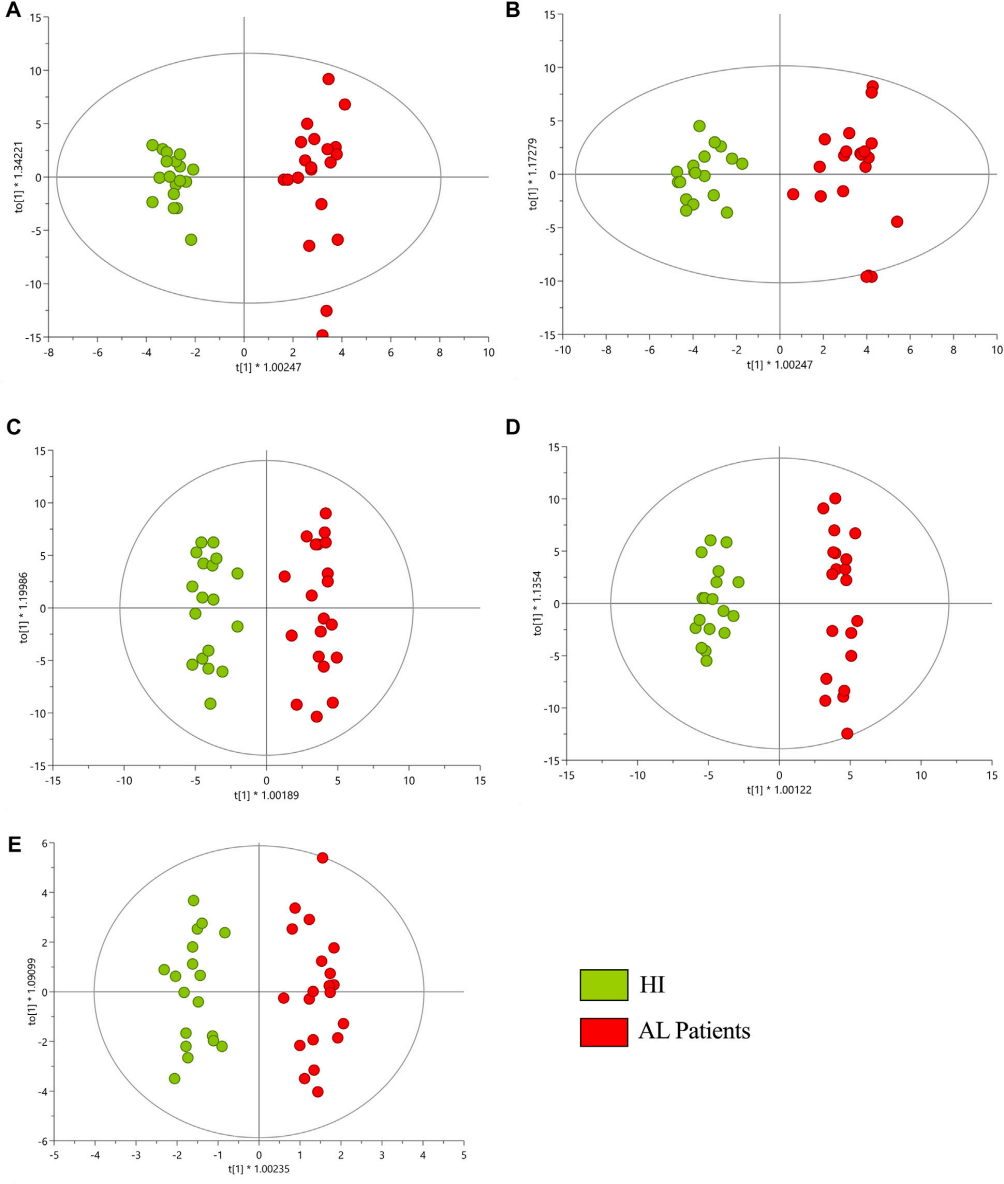
OPLS-DA score plot with Pareto scaling for a metabolic analysis of HI and AL patients. **(A)** GM-LC/MS (+) *R* ^2^: 0.958, Q^2^: 0.852, *p*_CV-ANOVA_: 1.948e^−11^. **(B)** GM-LC/MS (−) *R* ^2^: 0.925, Q^2^: 0.838, *p*_CV-ANOVA_: 7.3713e^−11^. **(C)** GL-LC/MS (+) *R* ^2^: 0.945, Q^2^: 0.670, *p*_CV-ANOVA_: 1.986e^−4^. **(D)** GL-LC/MS (−) *R* ^2^: 0.972, Q^2^: 0.789, *p*_CV-ANOVA_: 6.991e^−8^. **(E)** GC/MS *R* ^2^: 0.937, Q^2^: 0.743, *p*_CV-ANOVA_: 9.262e^−7^. Red dots correspond to AL patients, and green dots are HI.

Multiplatform metabolomic and lipidomic analyses of AL and HI plasma samples were conducted using different approaches aimed at detecting the largest possible number of metabolites. The performance of the different analytical platforms was evaluated by clustering the quality control (QC) samples using PCA models. In these models, a clear grouping of the QC samples belonging to each analytical platform was observed, indicating reliable, consistent performance and the conservation of biological variation across the platforms used (Supplementary Figure S1). Following the supervised OPLS-DA analyses, a discrimination between the HI group (green dots) and the AL patients (red dots) was observed for each platform, as depicted in Figure 1. This suggests distinct metabolomic profiles associated with the development of leukemia. The results indicated acceptable values ranging between 0.972 and 0.925 for *R* ^2^ and 0.670 and 0.852 for Q^2^ in the cross-validation test in the metabolomic and lipidomic analyses on all analytical platforms used (Wheelock and Wheelock, 2013). On the other hand, volcano plots were generated to show the metabolites that were significant [*p* < 0.05, Log2(FC) < 1.3] by univariate analysis (Supplementary Figure S2).

**FIGURE 2:**
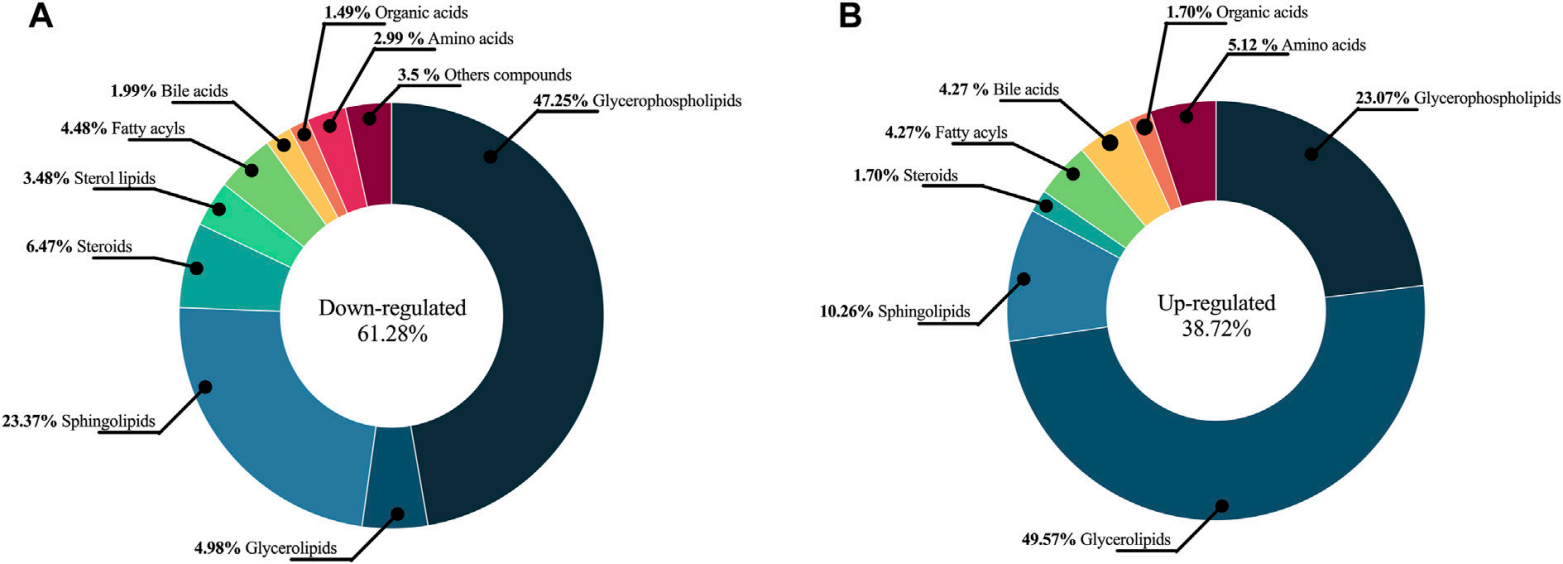
The chemical classes altered between AL patients and HI. The chemical classes and percentages are shown in a pie chart. **(A)** Downregulated metabolites **(B)** Upregulated metabolites.

A total of 328 metabolites differentially expressed between HI and AL were determined using a combination of MVA (VIP > 1 with JK), UVA (*p* < 0.05) applied on adjusted *p*-values, and fold change Log2(FC) > 1 and < 1). The detected metabolites during data processing across the different analytical techniques used are presented in Supplementary Table S2, and A typical metabolic fingerprint from each platform is presented in Supplementary Figures S3–S5. The compounds altered between AL patients and HI showed 61.28% (201 metabolites) downregulated metabolites and 38.72% (127 metabolites) upregulated metabolites. In respect of downregulated metabolites, we found glycerophospholipids (47.25%) to be the most representative, sphingolipids (23.37%), and a lower percentage (<7%) of sterol lipids, steroids, amino acids, bile acids, fatty acyls, organic acids, organooxygen compounds, and carnitines (Figure 2A). In the upregulated group, we observed that 47.25% of those metabolites were glycerophospholipids, 10.26% sphingolipids, 49.57% glycerolipids, 4.27% fatty acyls, and less than 8% corresponded to sterol lipids, amino acids, bile acids, organic acids, imidazopyrimidines, and steroids (Figure 2B).

**FIGURE 3:**
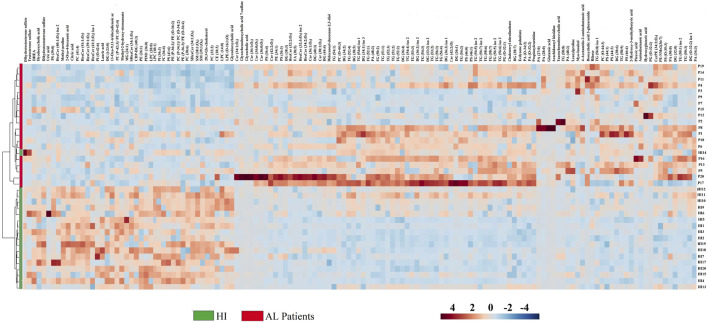
Heatmap of the metabolites and lipids with statistically significant variation. Fold Change ≥ 1.5 and ≤ 0.5, *p* < 0.05 between AL patients and HI.

For greater reliability, the Log2(FC) was adjusted to ≥ 1.5 and ≤ 0.5, and 135 metabolites. The set of altered metabolites (FC > 1.5 or < 0.5) between the two groups was analyzed using heatmaps, which enable the visualization of patterns of metabolite changes among the groups. Therefore, blue colors indicate decreased metabolite levels, while red colors indicate increased metabolite levels in AL patients (Figure 3). The clustering analysis in the heatmap reveals a clear grouping of samples from AL patients (green) and HI patients (red), indicating similarity in the metabolomic profiles among individuals within each group.

The most significant variations between AL patients and HI are observed in the glycerophospholipids group, which includes metabolites such as lysophosphatidylcholines, phosphatidylcholines (PCs), phosphatidylserine, phosphatidylethanolamine, lysophosphatidylethanolamines, and some sphingolipids like sphingomyelin. These metabolites were predominantly found to be downregulated in AL patients (represented by blue colors in the heatmap). In contrast, the glycerolipids group, including triacylglycerols (TG), diacylglycerols (DG), some organic acids like pyruvic acid and hydroxyglutaric acid, and amino acids such as 4-acetamido-amino butanoic acid, glutamic acid, amino butanoic acid, and leucylproline, showed trends towards upregulation in AL patients (represented by red colors in Figure 3). These results suggest significant alterations in biochemical metabolites, particularly lipid compounds, in the plasma of AL patients compared to the control group.

### Metabolic pathways associated with sphingolipid and glycerophospholipids are altered in AL patients

**FIGURE 4:**
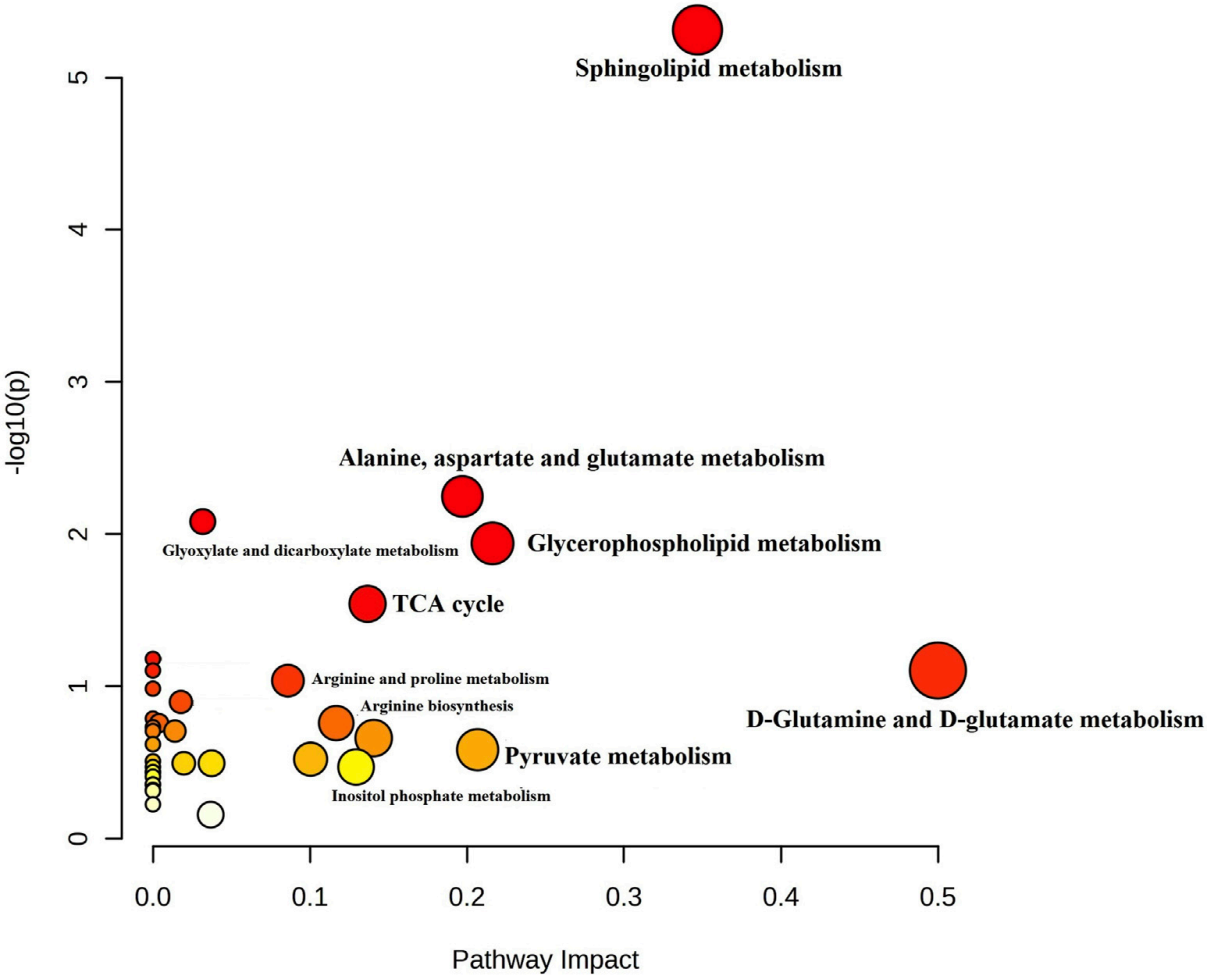
Summary of Metabolomic Pathway Analysis (MetPA) as generated by MetaboAnalyst 5.0 software package in AL patients. Using the KEGG database (All the matched pathways are displayed as circles). The color of each circle is based on *p*-values (darker colors indicate more significant changes of metabolites in the corresponding pathway). In contrast, the circle size corresponds to the pathway impact score. The most impacted pathways having high statistical significance scores are annotated (*p*-value < 0.05; pathway impact values ≥ 0.2.

**FIGURE 5:**
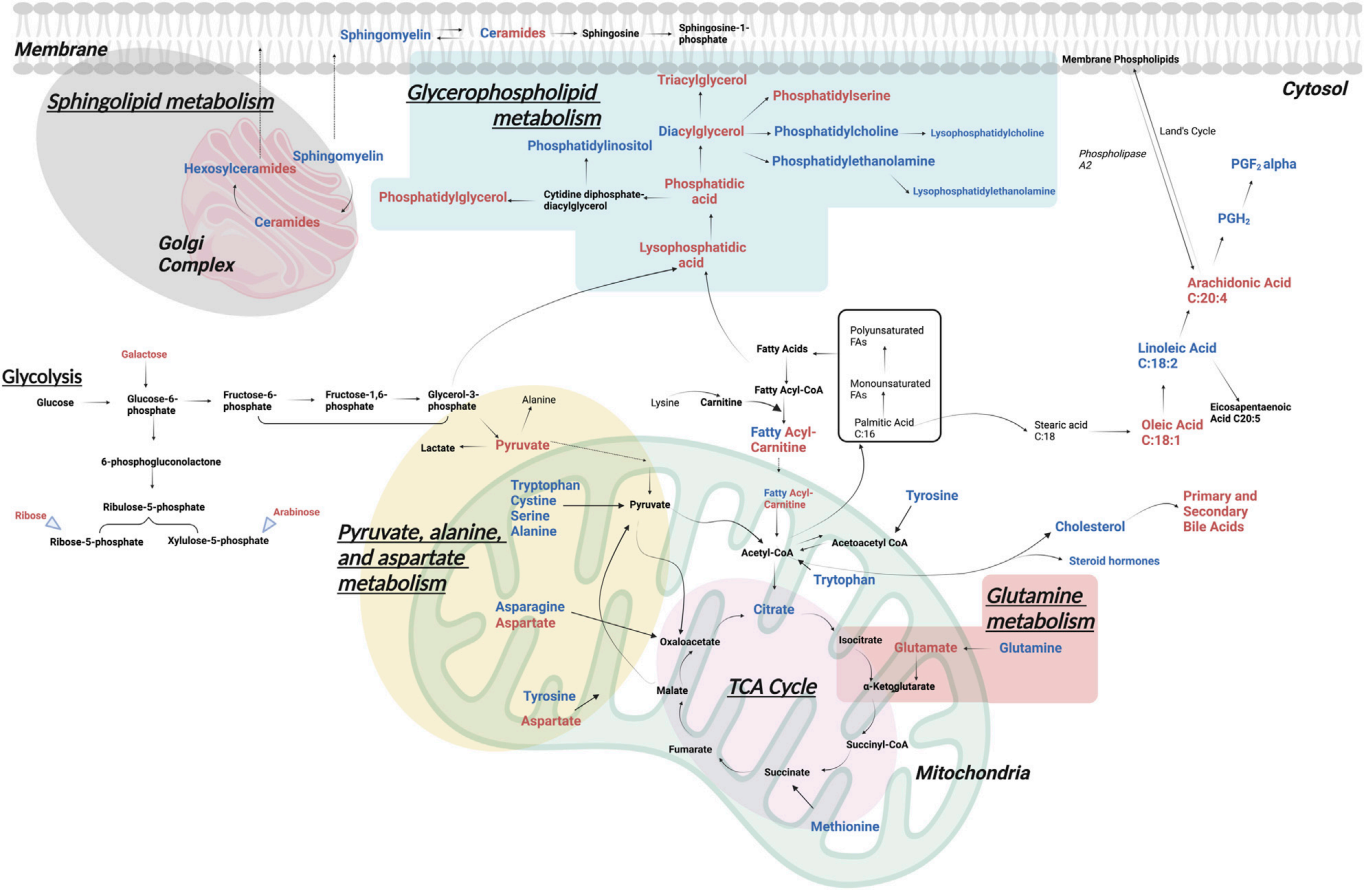
Significantly altered metabolomic pathways in AL. The main metabolic pathways altered in patients with AL vs. HI, identified by different metabolomic and lipidomic analytical platforms in peripheral blood plasma, are represented. In red upregulated and in blue downregulated metabolites.

For a better understanding of the metabolic dysregulation between the two groups, differential metabolites were imported into MetaboAnalyst 5.0 to perform the Metabolomic Pathway Analysis. The *x*-axis represents the pathway impact value computed from pathway topological analysis, and the *y*-axis is the-log of the *p*-value obtained from pathway enrichment analysis. The pathways that were most significantly changed are characterized by both a high-log(*p) value* and a high impact value (top right region). The node color of each pathway is determined by the *p*-value (red = lowest *p*-value and highest statistical significance), and the node radius (size) is based on the pathway impact factor, with the biggest indicating the highest impact (Mashabela et al., 2022). The metabolic pathways that were significantly altered in patients with AL compared with HI were sphingolipids, glycerophospholipids, alanine, aspartate, and glutamate metabolism (Figure 4). The metabolites identified within these altered pathways are a significant increase in pyruvate and AA and a significant decrease in linoleic acid, phosphatidylcholine, phosphatidylethanolamine, and lysophosphatidylcholine were found in the patients. A summary graph of the main altered 328 metabolites and their participation in the different metabolic pathways associated with leukemia is shown in Figure 5.

### The HI have higher TAC than the AL patients

**FIGURE 6:**
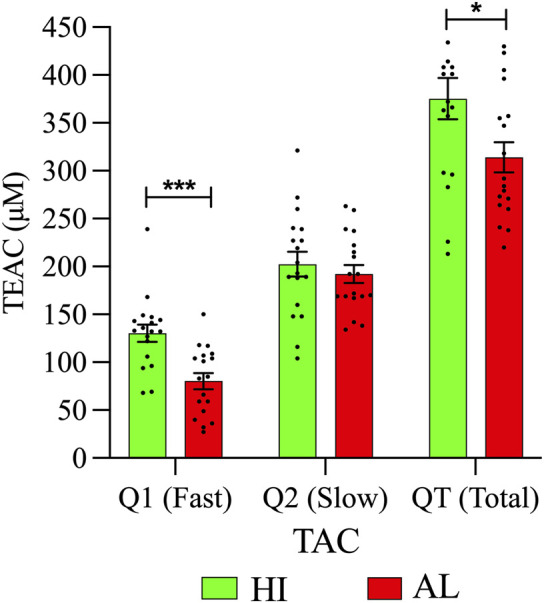
Plasma metabolic differences between AL patients and HI. TAC levels in the peripheral blood plasma of patients, and HI are expressed in Trolox Equivalent Antioxidant Capacity (TEAC). In all cases, data are represented as the mean ± SEM.

This work shows a higher concentration of slow antioxidants (Q2) than fast antioxidants (Q1), both in HI and in patients. Also, significant differences in rapid antioxidants (Q1) between HI and patients with AL were observed. Regarding the QT value, the patients presented less TAC than the HI (Figure 6; Supplementary Table S3). It is possible that there is a relationship between the decrease in TAC and the lipid alterations present in the patients. The decrease in antioxidant systems would favor an increase in ROS, which could stimulate survival signals or oxidize macromolecules such as lipids, inducing cell death (Barrera, 2012).

## Discussion

Thanks to the technical progress of metabolomics and the complexity of biological samples, the simultaneous use of several analytical platforms allows expanding the coverage of identification and characterization of metabolites, making it possible to find new non-invasive biomarkers for the diagnosis and prognosis of AL, as well as delving into the characteristics and biological differences of the lineage of origin of the disease, lymphoid or myeloid (Bruno et al., 2018). In this study, metabolic differences were analyzed at the plasma level between 18 HI and 20 AL patients, using metabolomics by LC-QTOF-MS and GC-QTOF-MS and lipidomics by LC-QTOF-MS.

Among the results obtained, it was shown that the primary metabolic alterations in patients with AL are related to lipid metabolism, in agreement with previous reports in the literature (Musharraf et al., 2016). Lipids are essential components of malignant tumors, as they are necessary for the growth and spread of the tumor. Fatty acids, cholesterol, and phospholipids are the most important sources of energy production, function as signaling molecules, and participate in the biogenesis of cell membranes. They can be provided by the tumor microenvironment or by cancer cells themselves through the activation of *de novo* synthesis pathways. Importantly, especially cells of the immune system, cancer-associated fibroblasts, and cancer-associated adipocytes, can also undergo changes in lipid content, hindering or promoting tumor aggressiveness (Fu et al., 2020; Vasseur and Guillaumond, 2022). In AML, lipids have been used to identify genetic signatures related to prognosis, the immunological panorama, and characteristics of the tumor microenvironment (Ding et al., 2022) and, in turn, as markers to predict the risk of acute graft-and-host disease (aGvHD) from allogeneic hematopoietic stem cell transplantation (alloHSCT) (Liu et al., 2019).

Fatty acids are the main building blocks of several lipid species, they can be channeled into various metabolic pathways to synthesize complex lipid species, including glycerolipids such as DG and TG, glycerophospholipids such as phosphatidic acid, phosphatidylethanolamine, PS, phosphatidylglycerol and phosphatidylcholine, sphingolipids and cholesterol (CL) including cholesterol ester (Koundouros and Poulogiannis, 2020). We observed a decrease in glycerophospholipids and an increase in glycerolipids in AL patients. Since glycerophospholipids are the main constituents of cell membranes, it is possible that they are being rapidly consumed by proliferating cells at the expense of Increased glycerolipids that serve as central intermediates in glycerophospholipid synthesis or as lipid storage molecules (Pan et al., 2021).

Within the increase in glycerolipids, TG was the most relevant. Altered glycerophospholipid metabolism has previously been associated with disease progression in pediatric ALL patients (Yunnuo et al., 2014) and an increase in TG in conjunction with a decrease in CL has been reported in both AML and ALL (Nahid et al., 2013), which has been related to a poor response to treatment (Guzmán and Sandoval, 2004). Particularly, Pabst et al. (2017) analyzed 20 samples from individuals with AML and 20 HI by GC-MS and ultraperformance liquid chromatography-electrospray ionization-quadrupole time-of-flight mass spectrometry (UPLC-ESI-QTOFMS), obtaining similar results, a decrease in PCs, cholesterol ester, and CL in the patients, however, they found a reduction in TG in the patients, probably associated with consumption by proliferating AML cells. From the point of view of the evolution of the disease, it has been described in other works, that patients with myelodysplastic syndromes who progressed to AL had higher TG levels than those who did not evolve (Qiao et al., 2022) and that in patients with ALL, after a 5-year disease-free period, they developed dyslipidemia with increased plasma TG, increased LDL CL, and decreased HDL CL, which was associated with an increased risk of atherosclerotic disease (Morel et al., 2017). These findings indicate that metabolic alterations at the lipid level with increased TG play an essential role in leukemogenesis, maintenance, and tumor progression but are also associated with clinical complications in these patients, such as atherosclerosis.

It should be noted that LICs or their equivalents in other cancer stem cells tumor models as initiating cells of the leukemogenesis process have a profile of genetic alterations associated with high risk and a specific metabolic profile, which are relevant as mechanisms implicated in treatment resistance and disease relapse (Marchand and Pinho, 2021). In particular, the increase in the synthesis of lipids in the LICs favors their self-renewal capacity by increasing the production of NADPH, which is an essential cofactor in reducing oxidized GSH to reduced GSH and in the maintenance of low ROS levels (Liu et al., 2022). Interestingly, Ito et al. (2012) showed that the PML gene controls asymmetric and symmetric HSC division through PPARδ activity, a regulator of fatty acid synthesis. In prostate cancer, a high lipid diet may accelerate tumor cell proliferation by increasing levels of insulin-like growth factor 1, IL-1α, IL-1β, IL -6, or TNF-α (Xu et al., 2014) or through activation of signaling pathways such as MCP-1/CCR2 (monocyte chemoattractant protein-1/C-C Motif Chemokine Receptor 2) (Huang et al., 2012). In addition, a high lipid diet accelerates the development of AML in a murine knock-in model for MLL-AF9 through the activation of the FLT3 receptor (Fms Related Receptor Tyrosine Kinase 3) on the membrane of c-KIT + primitive hematopoietic stem cells, with subsequent activation of the JAK3-STAT3 (Janus kinase/signal transducer and activator of transcription) signaling pathway (Hermetet et al., 2020).

Of the lipids, the ones best characterized in oncogenic signaling are phosphoinositols (PI) and ceramides/sphingolipids (SF), which we found decreased in patients compared to HI. However, this is one of the few studies that reflect this alteration at plasma levels in patients with AL (Calderon-Rodríguez et al., 2019). PIs are precursors of phosphoinositide, such as PI(3,4,5)P3, which can promote tumorigenesis by activating the AKT/mTORC1/2 pathway, which is frequently altered in AL (Nepstad et al., 2020), while SF participates in regular signals of cell survival or apoptosis (Ogretmen, 2018). Relevantly, the activation of cell signaling pathways due to mutations in oncogenes such as Ras and FLT3 plays a vital role in the metabolic reprogramming of leukemic blasts (Wojcicki et al., 2020). In fact, recent *in vitro* studies have shown that distinct genetic changes in AML are associated with improved dynamics and metabolism of different types of lipids, such as ceramides. Additionally, it has been found that patients with abnormal karyotypes, particularly those who have recurrent AML genetic changes like the t(8; 21)(q22; q22.1);*RUNX1-RUNX1T1* translocation or inv(16)(p13.1q22) inversion, have higher levels of ceramide/sphingolipid production (Stefanko et al., 2017).

The other altered metabolites were amino acids. Leucylproline, 4-acetamido-amino butanoic acid, glutamate, amino butanoic acid, and methylmethylproline were found to be increased in the patients. Leucylproline is a dipeptide formed by leucine and proline residues. On the one hand, leucine is part of the branched-chain amino acids (BCAA), which have been shown to be essential for the proliferation of leukemic cells (independent of their lineage). Since it supports the synthesis of non-essential amino acids and the TCA cycle (Tabe et al., 2019). Most patients with AML and ALL have a high level of BCAA transporters (BCAT1), while serum BCAA levels are reduced, suggesting active absorption of BCAA (Kikushige et al., 2023) and the formation of secondary metabolites (dipeptides). We found a reduction in glutamine and an increase in glutamate, suggesting an active metabolism of glutamine. Particularly, glutamate has been described as an exquisite source for leukemic cells since it promotes a tumor phenotype by participating in signaling reactions; it is a source of nitrogen for DNA synthesis and other amino acids; it participates in redox reactions through GSH; and it is a source of biomass and energy as it is incorporated into the TCA cycle (Kreitz et al., 2019). Carnitines are a fundamental part of the synthesis of fatty acids and are vital mediators for tumor metabolic plasticity (Melone et al., 2018). Like us, Morad et al. (2022) demonstrated a reduction in plasma O-acetyl carnitine in patients with ALL and AML. However, the metabolism of carnitines must be studied in depth because some chemotherapeutic drugs interfere with the absorption, synthesis, and excretion of carnitine in non-tumor tissues, leading to secondary carnitine deficiency and therefore multi-organ toxicity, which can be reversed with carnitine treatment without affects effectiveness. Anticancer by affects effectiveness anticancer (Sayed-Ahmed, 2010).

Tumor cells develop a mechanism where they adjust to the high ROS by expressing elevated levels of antioxidant proteins to detoxify them while maintaining pro-tumorigenic signaling and resistance to apoptosis. At the systemic level, a reduction in the expression of antioxidant enzymes and antioxidant capacity has been reported in AL samples (Rasool et al., 2015; Chaudhary et al., 2023). The reduction in TAC could reflect the consumption of endogenous antioxidants due to the generation of free radicals by the leukemic process (mutations or enzyme alterations) and maintain the redox balance The lower concentration of fast antioxidants (Q1) is expected given their oxidative potential. The increase in free radicals can cause lipid peroxidation, where polyunsaturated fatty acids are more susceptible, such as arachidonic acid. Iron-dependent lipid peroxidation is an important driver of ferroptosis, and ferroptosis is critically involved in the pathogenesis of AL. Interestingly, circulating antioxidants related to dietary intake (vitamin C, carotenoids, vitamin A, and vitamin E) may impact tumor progression in some types of cancer and vary depending on the dietary culture (Abenavoli et al., 2019; Yin et al., 2022).

We recognize that our study consolidates a low number of patients, however, there are not a huge number of primary acute leukemia samples in our region, which is reflected by the number of patients included in recent papers in Colombia (Calderon-Rodríguez et al., 2019) and in Latin America (Aguirre-Guillén et al., 2017). Therefore, our study is pioneering in a different social and cultural context and our findings may inspire more research on the metabolism of malignant hemopathies. We will verify the data in a larger cohort of patients in the medium term. Likewise, this metabolic profile will help us to follow the evolution of acute leukemia patients recruited in a clinical trial in which a new medicant directed to metabolism regulation is tested. We also consider integrating other omics techniques, particularly transcriptomics with metabolomics, to strengthen longitudinal studies, offering the opportunity to design and apply personalized treatments and advance in the search for biomarkers predictive of clinical response.

## Conclusion

This study reinforces previous observations of lipid abnormalities in patients with acute leukemia (AL), highlighting the significance of these metabolic dysregulations in the disease. Our findings indicate that glycerophospholipid metabolism, sphingolipid metabolism, and the metabolism of alanine, aspartate, glutamate, and glutamine are the primary deregulated pathways in AL patients. Additionally, we observed a lower total antioxidant capacity (TAC) in AL patients, reflecting the consumption of antioxidants during the leukemogenic process. These metabolic findings contribute to a deeper understanding of the physiological characteristics of leukemia and provide valuable insights for targeted therapeutic interventions and personalized treatment strategies. Furthermore, to our knowledge, this research represents the first metabolomics investigation conducted on the Colombian population, underscoring the novelty of our results. Future validation studies are necessary to confirm these findings with a larger cohort and elucidate their clinical implications.

## Data Availability

The original contributions presented in the study are included in the article/Supplementary Material, further inquiries can be directed to the corresponding authors.
