# Supplementary material for: Untargeted metabolomic and lipidomic analyses reveal lipid dysregulation in the plasma of acute leukemia patients
Source: Front Mol Biosci. 2023 Nov 10;10:1235160. doi: 10.3389/fmolb.2023.1235160 (PMC10667492; doi:10.3389/fmolb.2023.1235160)
Supplement: Supplementary file 1 [file DataSheet1.docx]

***Supplementary Material***

1. **Supplemental Tables**

| **SUPPLEMENTARY TABLE 1: CLINICAL CHARACTERISTICS OF THE PATIENTS INCLUDED IN THE STUDY AND CHARACTERISTICS OF THE HEALTHY INDIVIDUALS** | | | | | | | | | | | | | | | | | | | | | | |
| --- | --- | --- | --- | --- | --- | --- | --- | --- | --- | --- | --- | --- | --- | --- | --- | --- | --- | --- | --- | --- | --- | --- |
| **PATIENTS** | | | | | | | | | | | | | | | | | | | | **HEALTHY INDIVIDUALS** | | |
| **ID** | **AGE** | **GENDER** | **SUBTYPE LEUKEMIA** | **DIAGNOSTIC** | | | | | | | | | **CHEMOTHERAPY PROGRAM (INDUCTION)** | **END OF INDUCTION** | | | | | | **ID** | **AGE** | **GENDER** |
|  |  |  |  | **WBC/µL** | **ANC/µL** | **HB (g/dL)** | **PLATELET/µL** | **% TUMOR CELLS IN BONE MARROW** | **KARYOTYPE** | **GENETIC ALTERNATIONS** | **LDH (U/L)** | **RISK** |  | **RMD** | **WBC/µL** | **ANC/µL** | **Hb (g/dL)** | **PLATELET/µL** | **RESPONSE** |  |  |  |
| P-1 | 35 | Male | AML M3 | 5,500 | 200 | 11.2 | 12,600 | 89.6 | Abnormal | PML-RARA | 360 | Intermedium | AIDA - PETHEMA | N.E. | N.A. | N.A. | N.A. | N.A. | N.R.* | HI-1 | 33 | F |
| P-2 | 27 | Male | B-ALL | 57,400 | 574 | 11.1 | 12,600 | 84.0 | Normal | N.A. | 449 | High | PETHEMA | Positive | 2,600 | ≥1000 | 9.2 | 132,300 | N.R. | HI-2 | 23 | F |
| P-3 | 57 | Male | AML | 205,000 | 3,700 | 9.4 | 75,600 | 52.0 | Normal | NMP1 | 11,317 | Intermedium | 7x3 | N.E. | N.A. | N.A. | N.A. | N.A. | N.R.* | HI-3 | 30 | F |
| P-4 | 67 | Male | AML | 4,700 | 1,600 | 8.6 | 126,300 | 29.0 | Normal | N.A. | N.A. | High | 5-Azacitidine | N.E. | N.A. | N.A. | N.A. | N.A. | N.E. | HI-4 | 28 | F |
| P-5 | 65 | Female | B-ALL | 72,200 | 7,200 | 11.9 | 46,200 | 89.6 | Abnormal | t(9;22) | 1,021 | High | PETHEMA PH+ | Positive | 2,100 | ≥1000 | 9.3 | 426,500 | N.R. | HI-5 | 24 | F |
| P-6 | 76 | Female | B-ALL | 5,700 | 3,800 | 12.0 | 165,700 | 59.0 | Abnormal | Complex | 245 | High | PETHEMA OLD | Positive | 2,810 | ≥1000 | 10.4 | 168,000 | N.R. | HI-6 | 23 | F |
| P-7 | 62 | Male | AML | 3,200 | 1,250 | 3.2 | 63,500 | 28.0 | Abnormal | Monosomy 7 | 274 | High | 7x3 | Positive | 600 | <1000 | 8.3 | 62,500 | N.R. | HI-7 | 28 | F |
| P-8 | 36 | Male | B-ALL | 403,000 | 5,800 | 7.4 | 8,300 | 93.9 | No growth | N.A. | 795 | High | PETHEMA | Positive | 1,800 | ≥1000 | 10.8 | 150,300 | N.R. | HI-9 | 39 | M |
| P-9 | 47 | Female | B-ALL | 45,200 | 800 | 7.9 | 102,100 | 92.9 | Abnormal | t(9;22) | 664 | High | PETHEMA 2011 | Positive | 5,600 | ≥1000 | 8.8 | 15,500 | N.R. | HI-10 | 42 | M |
| P-10 | 50 | Female | AML | 42,400 | 17,300 | 8.4 | 100,800 | 29.0 | No growth | N.A. | 396 | High | 7x3 | N.E. | N.A. | N.A. | N.A. | N.A. | N.E. | HI-11 | 32 | M |
| P-11 | 49 | Female | AML | 401,800 | 4,000 | 10.0 | 29,400 | 93.7 | Normal | NMP1 | 1,235 | Intermedium | 7x3 | N.E. | N.A. | N.A. | N.A. | N.A. | N.R.* | HI-12 | 35 | F |
| P-12 | 46 | Female | AML | 3,900 | 195 | 10.0 | 228,000 | 20.0 | Abnormal | Complex | 330 | High | 5-Azacitidine | N.E. | N.A. | N.A. | N.A. | N.A. | N.E. | HI-13 | 25 | M |
| P-13 | 21 | Male | B-ALL | 162,800 | 9,768 | 10.8 | 79,800 | 89.7 | Abnormal | t(1;19) | 2,551 | High | PETHEMA 2011 | Negative | 7,800 | ≥1000 | 10.6 | 213,800 | R.C. | HI-14 | 54 | M |
| P-14 | 35 | Female | AML | 37,400 | 744 | 9.3 | 35,700 | 78.6 | Normal | N.A. | N.A. | Intermedium | 7x3 | Positive | 14,800 | ≥1000 | 8.7 | 358,800 | N.R. | HI-15 | 27 | F |
| P-15 | 51 | Female | AML | 27,700 | 270 | 11.5 | 67,500 | 69.5 | Abnormal | Complex | N.A. | High | 7x3 | Negative | 5,600 | ≥1000 | 9.2 | 326,800 | R.C. | HI-17 | 61 | F |
| P-16 | 44 | Female | B-ALL | 5,700 | 2,200 | 9.6 | 34,500 | 56.0 | Normal | N.A. | 453 | High | PETHEMA 2011 | Positive | 6,100 | ≥1000 | 10.6 | 330,900 | N.R. | HI-18 | 27 | M |
| P-17 | 21 | Female | Phenotype mixed | 35,000 | 700 | 9.1 | 103,800 | 84.2 | Normal | N.A. | 1,905 | High | PETHEMA 2011 | Positive | 8,500 | ≥1000 | 8.6 | 318,800 | N.R. | HI-19 | 19 | M |
| P-18 | 31 | Male | AML | 800 | 0 | 3.8 | 25,200 | 90.7 | Abnormal | N.A. | 124 | High | 7x3 | N.E. | N.A. | N.A. | N.A. | N.A. | N.E. | HI-20 | 25 | M |
| P-19 | 46 | Male | B-ALL | 10,600 | 0 | 5.5 | 10,500 | 93.1 | Abnormal | Complex | 527 | High | PETHEMA 2011 | Negative | 7,500 | <1000 | 9.4 | 226,800 | N.R. |  |  |  |
| P-20 | 51 | Female | B-ALL | 5,700 | 2,200 | 9.6 | 34,500 | 56.0 | Normal | N.A. | 453 | High | PETHEMA 2011 | Positive | 6,100 | ≥1000 | 10.6 | 330,900 | N.R. |  |  |  |
| WBC: White blood cells; AML M3: Promyelocytic - acute myeloid leukemia, AML: Acute myeloid leukemia; B-ALL: B-acute lymphoid leukemia; ANC: Absolute neutrophil count; Hb: Hemoglobin; LDH: Lactate dehydrogenase; RMD: Minimal residual disease by flow cytometry; N.E.: Not evaluated; N.A.: Not applicable or not available; N.R.: Not response; R.C.: Complete remission. HI: Healthy individuals. *Patients with premature death. | | | | | | | | | | | | | | | | | | | | | | |

| **^SUPPLEMENTARY TABLE 2. LIST OF ALL IDENTIFIED METABOLITES^** | | | | | | | | | | | | |  |
| --- | --- | --- | --- | --- | --- | --- | --- | --- | --- | --- | --- | --- | --- |
| **Compound** | **Formula** | **Mass** | **RT (min)** | **Error (ppm)** | **Adduct** | **^a^CV for QC (%)** | **Analytical platform** | **DET** | **^b^ID Level** | **^c^Fold Change** | ***^d^p* value** | **^e^VIP** |  |
| ***Glycerolipids*** | | | | | | | | | | | | |  |
| DG 27:0 | C_30_H_58_O_5_ | 498.4284 | 34.48 | 10 | [M+Na]^+^ | 6.58 | GM-RP-LC-QTOF-MS | ESI + | 3 | 0.59 | 2.00E-03ⱡ | 1.27 |  |
| DG i-23:0 | C_26_H_50_O_5_ | 442.3658 | 29.37 | 0 | [M-H]^-^ | 12.38 | GM-RP-LC-QTOF-MS | ESI - | 3 | 0.47 | 2.29E-03ⱡ | 1.25 |  |
| MG 24:1 | C_27_H_52_O_4_ | 440.3866 | 30.27 | 0 | [M+HCOOH-H]^-^ | 8.11 | GM-RP-LC-QTOF-MS | ESI - | 3 | 0.35 | 6.36E-05ⱡ | 1.49 |  |
| DG 32:0 | C_35_H_68_O_5_ | 568.5067 | 26.84 | 5 | [M+H-H_2_O]^+^ | 2.18 | GL-RP-LC-QTOF-MS | ESI + | 3 | 2.26 | 4.19E-03ⱡ | 1.44 |  |
| DG 33:2 iso1 | C_37_H_68_O_5_ | 592.5067 | 26.18 | 5 | [M+H-H_2_O]^+^ | 2.10 | GL-RP-LC-QTOF-MS | ESI + | 4 | 1.93 | 1.89E-02ⱡ | 1.35 |  |
| DG 33:2 iso2 | C_37_H_68_O_5_ | 592.5067 | 26.49 | 5 | [M+H-H_2_O]^+^ | 1.12 | GL-RP-LC-QTOF-MS | ESI + | 4 | 1.98 | 2.94E-03ⱡ | 1.44 |  |
| DG 34:1 iso1 | C_37_H_70_O_5_ | 594.5223 | 26.21 | 4 | [M+H-H_2_O]^+^ | 4.29 | GL-RP-LC-QTOF-MS | ESI + | 3 | 2.00 | 1.77E-02ⱡ | 1.38 |  |
| DG 34:1 iso2 | C_37_H_70_O_5_ | 594.5223 | 26.48 | 5 | [M+H-H_2_O]^+^ | 1.83 | GL-RP-LC-QTOF-MS | ESI + | 3 | 1.99 | 2.54E-02ⱡ | 1.45 |  |
| DG 34:1 iso3 | C_37_H_70_O_5_ | 594.5223 | 26.84 | 5 | [M+H-H_2_O]^+^ | 2.20 | GL-RP-LC-QTOF-MS | ESI + | 3 | 2.48 | 3.69E-02ⱡ | 1.53 |  |
| DG 34:2 | C_37_H_68_O_5_ | 592.5067 | 25.85 | 4 | [M+H-H_2_O]^+^ | 1.90 | GL-RP-LC-QTOF-MS | ESI + | 3 | 1.58 | 1.26E-01ⱡ | 1.19 |  |
| DG 36:1 | C_39_H_74_O_5_ | 622.5536 | 28.44 | 4 | [M+H-H_2_O]^+^ | 2.20 | GL-RP-LC-QTOF-MS | ESI + | 3 | 2.36 | 1.55E-02ⱡ | 1.47 |  |
| DG 36:2 iso 1 | C_39_H_72_O_5_ | 620.5380 | 27.20 | 6 | [M+H-H_2_O]^+^ | 1.98 | GL-RP-LC-QTOF-MS | ESI + | 3 | 2.43 | 1.55E-02ⱡ | 1.61 |  |
| DG 36:2 iso 2 | C_39_H_72_O_5_ | 620.5380 | 27.58 | 5 | [M+H-H_2_O]^+^ | 5.04 | GL-RP-LC-QTOF-MS | ESI + | 3 | 2.46 | 7.66E-04ⱡ | 1.55 |  |
| DG 36:3 iso 1 | C_39_H_70_O_5_ | 618.5223 | 26.48 | 5 | [M+H-H_2_O]^+^ | 1.45 | GL-RP-LC-QTOF-MS | ESI + | 3 | 1.98 | 3.66E-03ⱡ | 1.46 |  |
| DG 36:3 iso 2 | C_39_H_70_O_5_ | 618.5223 | 26.83 | 5 | [M+H-H_2_O]^+^ | 2.28 | GL-RP-LC-QTOF-MS | ESI + | 4 | 2.05 | 1.99E-03ⱡ | 1.42 |  |
| DG 36:3 iso 3 | C_39_H_70_O_5_ | 618.5223 | 26.16 | 5 | [M+H-H_2_O]^+^ | 1.37 | GL-RP-LC-QTOF-MS | ESI + | 4 | 1.56 | 1.21E-02ⱡ | 1.13 |  |
| DG 36:4 | C_39_H_68_O_5_ | 616.5067 | 25.86 | 5 | [M+H-H_2_O]^+^ | 1.89 | GL-RP-LC-QTOF-MS | ESI + | 4 | 1.55 | 1.73E-02ⱡ | 1.18 |  |
| DG 38:3 | C_41_H_74_O_5_ | 646.5536 | 16.13 | 6 | [M-H-H_2_O]^-^ | 3.38 | GL-RP-LC-QTOF-MS | ESI - | 4 | 2.91 | 4.23E-05ⱡ | 1.87 |  |
| DG 38:5 | C_41_H_70_O_5_ | 642.5223 | 17.78 | 1 | [M+HCOOH-H]^-^ | 13.83 | GL-RP-LC-QTOF-MS | ESI - | 3 | 2.14 | 7.50E-03ⱡ | 1.40 |  |
| DG 38:6 | C_41_H_68_O_5_ | 640.5070 | 16.69 | 1 | [M+HCOOH-H]^-^ | 19.35 | GL-RP-LC-QTOF-MS | ESI - | 3 | 2.02 | 2.59E-02ⱡ | 1.27 |  |
| DG 38:7 | C_41_H_66_O_5_ | 638.4910 | 16.65 | 0 | [M+H]^+^ | 19.06 | GL-RP-LC-QTOF-MS | ESI + | 3 | 1.54 | 1.19E-02ⱡ | 1.13 |  |
| DG 40:3 | C_43_H_78_O_5_ | 674.5849 | 17.95 | 5 | [M-H-H_2_O]^-^ | 4.73 | GL-RP-LC-QTOF-MS | ESI - | 4 | 1.88 | 7.29E-04ⱡ | 1.30 |  |
| DG 41:3 | C_44_H_80_O_5_ | 688.6006 | 18.99 | 7 | [M-H-H_2_O]^-^ | 2.46 | GL-RP-LC-QTOF-MS | ESI - | 3 | 0.71 | 5.69E-03ⱡ | 1.10 |  |
| DG 44:2 | C_47_H_88_O_5_ | 732.6632 | 21.34 | 5 | [M-H-H_2_O]^-^ | 10.62 | GL-RP-LC-QTOF-MS | ESI - | 3 | 0.59 | 8.49E-03ⱡ | 1.30 |  |
| DG 44:4 | C_47_H_84_O_5_ | 728.6319 | 19.80 | 5 | [M-H-H_2_O]^-^ | 3.01 | GL-RP-LC-QTOF-MS | ESI - | 3 | 1.63 | 2.26E-03ⱡ | 1.21 |  |
| DG 46:3 | C_49_H_90_O_5_ | 758.6788 | 22.09 | 4 | [M-H-H_2_O]^-^ | 3.85 | GL-RP-LC-QTOF-MS | ESI - | 3 | 0.72 | 1.40E-02ⱡ | 1.00 |  |
| DG O-32:2 iso 1 | C_35_H_66_O_4_ | 550.4961 | 26.17 | 5 | [M+H]^+^ | 2.40 | GL-RP-LC-QTOF-MS | ESI + | 3 | 1.76 | 1.25E-02ⱡ | 1.24 |  |
| DG O-32:2 iso 2 | C_35_H_66_O_4_ | 550.4961 | 26.50 | 6 | [M+H]^+^ | 2.11 | GL-RP-LC-QTOF-MS | ESI + | 3 | 1.61 | 3.39E-02ⱡ | 1.05 |  |
| TG 50:1 iso 1 | C_53_H_100_O_6_ | 832.7520 | 25.86 | 6 | [M+Na]^+^ | 7.32 | GL-RP-LC-QTOF-MS | ESI + | 3 | 1.64 | 2.21E-02ⱡ | 1.25 |  |
| TG 50:1 iso 2 | C_53_H_100_O_6_ | 832.7520 | 26.85 | 5 | [M+NH_4_]^+^ | 1.94 | GL-RP-LC-QTOF-MS | ESI + | 2 | 2.29 | 3.74E-03ⱡ | 1.52 |  |
| TG 50:2 | C_53_H_98_O_6_ | 830.7363 | 26.19 | 5 | [M+NH_4_]^+^ | 2.19 | GL-RP-LC-QTOF-MS | ESI + | 2 | 1.75 | 2.06E-02ⱡ | 1.32 |  |
| TG 50:3 | C_53_H_96_O_6_ | 828.7207 | 25.59 | 5 | [M+Na]^+^ | 1.05 | GL-RP-LC-QTOF-MS | ESI + | 3 | 1.52 | 3.39E-02ⱡ | 1.09 |  |
| TG 51:1 | C_54_H_102_O_6_ | 846.7676 | 27.38 | 5 | [M+NH_4_]^+^ | 3.48 | GL-RP-LC-QTOF-MS | ESI + | 3 | 1.57 | 2.22E-02ⱡ | 1.09 |  |
| TG 51:2 | C_54_H_100_O_6_ | 844.7520 | 26.67 | 5 | [M+NH_4_]^+^ | 4.40 | GL-RP-LC-QTOF-MS | ESI + | 3 | 1.53 | 3.84E-02ⱡ | 1.14 |  |
| TG 51:5 | C_54_H_94_O_6_ | 838.7050 | 19.87 | 5 | [M+Cl]^-^ | 4.38 | GL-RP-LC-QTOF-MS | ESI - | 4 | 0.58 | 2.35E-04ⱡ | 1.31 |  |
| TG 52:1 | C_55_H_104_O_6_ | 860.7833 | 28.01 | 5 | [M+Na]^+^ | 1.90 | GL-RP-LC-QTOF-MS | ESI + | 2 | 1.94 | 5.45E-03ⱡ | 1.31 |  |
| TG 52:2 | C_55_H_102_O_6_ | 858.7676 | 27.20 | 5 | [M+NH_4_]^+^ | 1.06 | GL-RP-LC-QTOF-MS | ESI + | 2 | 1.69 | 1.81E-03ⱡ | 1.34 |  |
| TG 52:3 | C_55_H_100_O_6_ | 856.7520 | 26.49 | 5 | [M+NH_4_]^+^ | 1.23 | GL-RP-LC-QTOF-MS | ESI + | 2 | 1.62 | 2.94E-03ⱡ | 1.28 |  |
| TG 52:4 | C_55_H_98_O_6_ | 854.7363 | 25.86 | 5 | [M+NH_4_]^+^ | 1.94 | GL-RP-LC-QTOF-MS | ESI + | 2 | 1.54 | 1.72E-02ⱡ | 1.23 |  |
| TG 53:2 | C_56_H_104_O_6_ | 872.7833 | 27.77 | 5 | [M+NH_4_]^+^ | 6.06 | GL-RP-LC-QTOF-MS | ESI + | 3 | 1.90 | 3.43E-03ⱡ | 1.35 |  |
| TG 53:3 | C_56_H_102_O_6_ | 870.7676 | 27.00 | 5 | [M+NH_4_]^+^ | 3.02 | GL-RP-LC-QTOF-MS | ESI + | 4 | 1.67 | 1.16E-02ⱡ | 1.25 |  |
| TG 53:4 | C_56_H_100_O_6_ | 868.7520 | 26.32 | 4 | [M+NH_4_]^+^ | 1.89 | GL-RP-LC-QTOF-MS | ESI + | 3 | 1.34 | 3.39E-02ⱡ | - |  |
| TG 54:1 | C_57_H_108_O_6_ | 888.8146 | 29.43 | 5 | [M+Na]^+^ | 6.70 | GL-RP-LC-QTOF-MS | ESI + | 3 | 1.60 | 3.25E-02ⱡ | 1.02 |  |
| TG 54:2 | C_57_H_106_O_6_ | 886.7989 | 28.44 | 5 | [M+NH_4_]^+^ | 1.78 | GL-RP-LC-QTOF-MS | ESI + | 3 | 2.68 | 1.55E-03ⱡ | 1.59 |  |
| TG 54:3 | C_57_H_104_O_6_ | 884.7833 | 27.58 | 5 | [M+NH_4_]^+^ | 3.00 | GL-RP-LC-QTOF-MS | ESI + | 2 | 2.32 | 7.10E-04ⱡ | 1.53 |  |
| TG 54:4 iso 1 | C_57_H_102_O_6_ | 882.7676 | 26.48 | 6 | [M+NH_4_]^+^ | 2.18 | GL-RP-LC-QTOF-MS | ESI + | 4 | 1.81 | 1.81E-03ⱡ | 1.38 |  |
| TG 54:4 iso 2 | C_57_H_102_O_6_ | 882.7676 | 26.83 | 5 | [M+NH_4_]^+^ | 2.61 | GL-RP-LC-QTOF-MS | ESI + | 3 | 2.05 | 3.66E-03ⱡ | 1.48 |  |
| TG 54:5 iso 1 | C_57_H_100_O_6_ | 880.7520 | 26.17 | 5 | [M+NH_4_]^+^ | 3.21 | GL-RP-LC-QTOF-MS | ESI + | 3 | 1.55 | 3.39E-02ⱡ | 1.17 |  |
| TG 54:5 iso 2 | C_57_H_100_O_6_ | 880.7520 | 26.45 | 5 | [M+NH_4_]^+^ | 3.72 | GL-RP-LC-QTOF-MS | ESI + | 3 | 2.62 | 5.18E-04ⱡ | 1.69 |  |
| TG 54:6 iso 1 | C_57_H_98_O_6_ | 878.7363 | 25.80 | 5 | [M+Na]^+^ | 1.05 | GL-RP-LC-QTOF-MS | ESI + | 2 | 1.60 | 2.79E-03ⱡ | 1.15 |  |
| TG 54:6 iso 2 | C_57_H_98_O_6_ | 878.7363 | 23.77 | 5 | [M-H]^-^ | 1.45 | GL-RP-LC-QTOF-MS | ESI - | 3 | 1.56 | 6.40E-03ⱡ | 1.05 |  |
| TG 54:11 | C_57_H_88_O_6_ | 868.6581 | 19.24 | 8 | [M+HCOOH-H]^-^ | 1.50 | GL-RP-LC-QTOF-MS | ESI - | 4 | 0.62 | 5.82E-05ⱡ | 1.31 |  |
| TG 55:11 | C_58_H_90_O_6_ | 882.6737 | 19.98 | 7 | [M+HCOOH-H]^-^ | 5.02 | GL-RP-LC-QTOF-MS | ESI - | 4 | 0.71 | 3.66E-04ⱡ | 1.10 |  |
| TG 56:3 | C_59_H_108_O_6_ | 912.8146 | 28.85 | 4 | [M+Na]^+^ | 2.86 | GL-RP-LC-QTOF-MS | ESI + | 3 | 1.67 | 1.60E-02ⱡ | 1.12 |  |
| TG 56:4 | C_59_H_106_O_6_ | 910.7989 | 27.93 | 5 | [M+Na]^+^ | 0.73 | GL-RP-LC-QTOF-MS | ESI + | 3 | 1.54 | 1.13E-02ⱡ | 1.07 |  |
| TG 56:5 iso 1 | C_59_H_104_O_6_ | 908.7833 | 27.24 | 4 | [M+NH_4_]^+^ | 1.84 | GL-RP-LC-QTOF-MS | ESI + | 3 | 2.05 | 5.29E-03ⱡ | 1.45 |  |
| TG 56:5 iso 2 | C_59_H_104_O_6_ | 908.7833 | 27.53 | 4 | [M+NH_4_]^+^ | 2.82 | GL-RP-LC-QTOF-MS | ESI + | 3 | 3.02 | 2.34E-05ⱡ | 1.86 |  |
| TG 56:6 iso 1 | C_59_H_102_O_6_ | 906.7676 | 26.56 | 4 | [M+NH_4_]^+^ | 3.62 | GL-RP-LC-QTOF-MS | ESI + | 3 | 1.91 | 3.39E-03ⱡ | 1.47 |  |
| TG 56:6 iso 2 | C_59_H_102_O_6_ | 906.7676 | 26.78 | 4 | [M+NH_4_]^+^ | 2.84 | GL-RP-LC-QTOF-MS | ESI + | 3 | 2.78 | 3.46E-05ⱡ | 1.78 |  |
| TG 56:7 iso 1 | C_59_H_100_O_6_ | 904.7520 | 26.11 | 5 | [M+Na]^+^ | 0.77 | GL-RP-LC-QTOF-MS | ESI + | 3 | 1.73 | 4.99E-04ⱡ | 1.32 |  |
| TG 56:7 iso 2 | C_59_H_100_O_6_ | 904.7520 | 26.23 | 4 | [M+NH_4_]^+^ | 1.36 | GL-RP-LC-QTOF-MS | ESI + | 3 | 1.89 | 4.51E-03ⱡ | 1.40 |  |
| TG 56:8 iso 1 | C_59_H_98_O_6_ | 902.7363 | 25.64 | 5 | [M+Na]^+^ | 2.00 | GL-RP-LC-QTOF-MS | ESI + | 3 | 1.75 | 3.66E-03ⱡ | 1.30 |  |
| TG 56:8 iso 2 | C_59_H_100_O_5_ | 888.7571 | 27.78 | 5 | [M+Na]^+^ | 3.65 | GL-RP-LC-QTOF-MS | ESI + | 3 | 1.53 | 8.62E-03ⱡ | 1.15 |  |
| TG 58:6 | C_61_H_106_O_6_ | 934.7989 | 27.66 | 4 | [M+Na]^+^ | 1.96 | GL-RP-LC-QTOF-MS | ESI + | 3 | 1.75 | 4.07E-04ⱡ | 1.30 |  |
| TG 58:7 | C_61_H_104_O_6_ | 932.7833 | 27.23 | 4 | [M+NH_4_]^+^ | 3.93 | GL-RP-LC-QTOF-MS | ESI + | 3 | 2.55 | 1.75E-04ⱡ | 1.66 |  |
| TG 58:8 | C_61_H_102_O_6_ | 930.7676 | 26.56 | 5 | [M+Na]^+^ | 2.21 | GL-RP-LC-QTOF-MS | ESI + | 3 | 1.81 | 1.19E-03ⱡ | 1.29 |  |
| TG 58:9 | C_61_H_100_O_6_ | 928.7520 | 25.93 | 4 | [M+Na]^+^ | 0.82 | GL-RP-LC-QTOF-MS | ESI + | 3 | 1.76 | 6.63E-03ⱡ | 1.14 |  |
| TG 62:7 | C_65_H_114_O_5_ | 974.8666 | 26.70 | 5 | [M-H]^-^ | 7.17 | GL-RP-LC-QTOF-MS | ESI - | 3 | 0.62 | 1.78E-03ⱡ | 1.20 |  |
| ***Glycerophospholipids*** | | | | | | | | | | | | |  |
| CDP-DG 40:3 | C_52_H_91_N_3_O_15_P_2_ | 1059.5925 | 20.19 | 4 | [M+NH_4_]^+^ | 3.83 | GM-RP-LC-QTOF-MS | ESI + | 4 | 0.47 | 1.00E-03ⱡ | 2.39 |  |
| CL 45:0 | C_54_H_104_O_17_P_2_ | 1086.6749 | 21.73 | 9 | [M-H]^-^ | 14.59 | GM-RP-LC-QTOF-MS | ESI - | 4 | 0.61 | 1.30E-02ⱡ | 1.45 |  |
| LPC 0:0/14:0 | C_22_H_46_NO_7_P | 467.3012 | 16.83 | 5 | [M+H]^+^ | 3.78 | GM-RP-LC-QTOF-MS | ESI +/- | 2 | 0.47 | 1.00E-03ⱡ | 2.11 |  |
| LPC 14:0/0:0 | C_22_H_46_NO_7_P | 467.3012 | 17.58 | 5 | [M+H]^+^ | 3.11 | GM-RP-LC-QTOF-MS | ESI +/- | 2 | 0.43 | 1.00E-03ⱡ | 2.42 |  |
| LPC 15:0 | C_23_H_48_NO_7_P | 481.3168 | 19.60 | 1 | [M+HCOOH-H]^-^ | 5.58 | GM-RP-LC-QTOF-MS | ESI - | 2 | 0.57 | 2.52E-04ⱡ | 1.58 |  |
| LPC 0:0/16:0 | C_24_H_50_NO_7_P | 495.3325 | 20.91 | 6 | [M+H]^+^ | 1.61 | GM-RP-LC-QTOF-MS | ESI +/- | 2 | 0.74 | 1.14E-03ⱡ | 1.55 |  |
| LPC 16:0/0:0 | C_24_H_50_NO_7_P | 495.3325 | 21.25 | 1 | [M+HCOOH-H]^-^ | 11.77 | GM-RP-LC-QTOF-MS | ESI - | 2 | 0.59 | 6.36E-04ⱡ | 1.47 |  |
| LPC 0:0/17:0 | C_25_H_52_NO_7_P | 509.3481 | 23.43 | 2 | [M+HCOOH-H]^-^ | 7.13 | GM-RP-LC-QTOF-MS | ESI +/- | 2 | 0.55 | 6.36E-04ⱡ | 1.58 |  |
| LPC 17:0/0:0 | C_25_H_52_NO_7_P | 509.3481 | 23.92 | 4 | [M+HCOOH-H]^-^ | 7.73 | GM-RP-LC-QTOF-MS | ESI +/- | 2 | 0.72 | 1.89E-02ⱡ | 1.24 |  |
| LPC 18:0/0:0 | C_26_H_54_NO_7_P | 523.3638 | 26.24 | 5 | [M+H]^+^ | 2.62 | GM-RP-LC-QTOF-MS | ESI + | 2 | 0.74 | 4.70E-02ⱡ | 1.49 |  |
| LPC 0:0/18:0 | C_26_H_54_NO_7_P | 523.3638 | 25.37 | 5 | [M+H]^+^ | 4.32 | GM-RP-LC-QTOF-MS | ESI +/- | 2 | 0.73 | 2.10E-02 | 1.49 |  |
| LPC O 18:0 | C_26_H_56_NO_6_P | 509.3845 | 27.45 | 5 | [M+H]^+^ | 6.23 | GM-RP-LC-QTOF-MS | ESI + | 2 | 0.71 | 1.30E-02ⱡ | 1.33 |  |
| LPC O 17:1;O | C_25_H_52_NO_7_P | 509.3481 | 23.34 | 1 | [M+HCOOH-H]^-^ | 8.41 | GM-RP-LC-QTOF-MS | ESI - | 3 | 0.51 | 1.33E-04ⱡ | - |  |
| LPC 18:1 | C_26_H_52_NO_7_P | 521.3481 | 23.35 | 5 | [M+H]^+^ | 6.03 | GM-RP-LC-QTOF-MS | ESI + | 2 | 0.49 | 1.00E-03ⱡ | 2.01 |  |
| LPC 0:0/18:2 | C_26_H_50_NO_7_P | 519.3325 | 19.52 | 5 | [M+H]^+^ | 4.59 | GM-RP-LC-QTOF-MS | ESI +/- | 2 | 0.53 | 1.00E-03ⱡ | 1.91 |  |
| LPC 18:2/0:0 | C_26_H_50_NO_7_P | 519.3325 | 20.19 | 5 | [M+H]^+^ | 1.41 | GM-RP-LC-QTOF-MS | ESI +/- | 2 | 0.59 | 1.00E-03ⱡ | 1.88 |  |
| LPC 0:0/18:3 | C_26_H_48_NO_7_P | 517.3168 | 18.11 | 4 | [M+H]^+^ | 7.01 | GM-RP-LC-QTOF-MS | ESI +/- | 2 | 0.48 | 1.00E-03ⱡ | 2.21 |  |
| LPC 18:3/0:0 | C_26_H_48_NO_7_P | 517.3168 | 18.47 | 5 | [M+H]^+^ | 10.50 | GM-RP-LC-QTOF-MS | ESI +/- | 2 | 0.62 | 1.47E-02 | 2.23 |  |
| LPC 20:0 | C_28_H_58_NO_7_P | 551.3951 | 31.15 | 4 | [M+H]^+^ | 5.94 | GM-RP-LC-QTOF-MS | ESI + | 2 | 0.42 | 1.00E-03ⱡ | 2.39 |  |
| LPC 20:2 | C_28_H_54_NO_7_P | 547.3638 | 24.01 | 5 | [M+H]^+^ | 7.63 | GM-RP-LC-QTOF-MS | ESI + | 2 | 0.64 | 4.70E-02ⱡ | 1.67 |  |
| LPC 0:0/20:3 | C_28_H_52_NO_7_P | 545.3481 | 21.11 | 4 | [M+H]^+^ | 8.52 | GM-RP-LC-QTOF-MS | ESI +/- | 2 | 0.64 | 2.30E-02ⱡ | 1.58 |  |
| LPC 20:3/0:0 | C_28_H_52_NO_7_P | 545.3481 | 21.76 | 5 | [M+H]^+^ | 5.99 | GM-RP-LC-QTOF-MS | ESI +/- | 2 | 0.63 | 2.00E-02ⱡ | 1.73 |  |
| LPC 20:5 | C_28_H_48_NO_7_P | 541.3168 | 18.27 | 4 | [M+H]^+^ | 5.30 | GM-RP-LC-QTOF-MS | ESI + | 2 | 0.71 | 1.84E-02 | 1.43 |  |
| LPE 18:0/0:0 | C_23_H_48_NO_7_P | 481.3168 | 23.80 | 2 | [M-H-H_2_O]^-^ | 14.70 | GM-RP-LC-QTOF-MS | ESI - | 2 | 0.54 | 1.02E-03ⱡ | - |  |
| LPE 0:0/18:2 | C_23_H_44_NO_7_P | 477.2855 | 19.34 | 4 | [M+H]^+^ | 4.85 | GM-RP-LC-QTOF-MS | ESI +/- | 2 | 0.68 | 1.19E-02 | 1.57 |  |
| LPE 18:2/:0:0 | C_23_H_44_NO_7_P | 477.2855 | 20.01 | 4 | [M+H]^+^ | 3.32 | GM-RP-LC-QTOF-MS | ESI +/- | 2 | 0.68 | 2.08E-02 | 1.61 |  |
| LPE 22:5 | C_27_H_46_NO_7_P | 527.3012 | 21.75 | 2 | [M-H]^-^ | 12.55 | GM-RP-LC-QTOF-MS | ESI - | 2 | 0.70 | 2.18E-02ⱡ | 1.15 |  |
| LPE O 16:1;0 | C_21_H_44_NO_7_P | 453.2855 | 17.57 | 3 | [M-H]^-^ | 6.46 | GM-RP-LC-QTOF-MS | ESI - | 3 | 0.46 | 1.96E-04ⱡ | 1.90 |  |
| LPE O 18:1 | C_23_H_48_NO_6_P | 465.3219 | 27.34 | 4 | [M+H]^+^ | 8.32 | GM-RP-LC-QTOF-MS | ESI +/- | 3 | 0.69 | 4.70E-02ⱡ | 1.12 |  |
| PA O 34:2 | C_37_H_71_O_7_P | 658.4937 | 18.76 | 4 | [M+H]^+^ | 14.89 | GL-RP-LC-QTOF-MS | ESI + | 2 | 1.78 | 8.80E-04ⱡ | 1.32 |  |
| PA 27:0 | C_30_H_59_O_8_P | 578.3948 | 9.60 | 7 | [M+Cl]^-^ | 8.50 | GM-RP-LC-QTOF-MS | ESI - | 4 | 6.33 | 2.60E-05ⱡ | 2.31 |  |
| PA 28:0 | C_31_H_61_O_8_P | 592.4104 | 10.89 | 5 | [M+Cl]^-^ | 6.63 | GM-RP-LC-QTOF-MS | ESI - | 4 | 4.66 | 1.20E-04ⱡ | 1.98 |  |
| PA 34:3 | C_37_H_69_O_7_P | 656.4781 | 17.74 | 4 | [M+H]^+^ | 17.10 | GL-RP-LC-QTOF-MS | ESI + | 3 | 1.68 | 1.81E-03ⱡ | 1.26 |  |
| PA 42:4 | C_45_H_81_O_8_P | 780.5669 | 16.00 | 5 | [M+NH_4_]^+^ | 3.16 | GL-RP-LC-QTOF-MS | ESI + | 3 | 0.61 | 2.65E-04ⱡ | 1.30 |  |
| PA 44:7 | C_47_H_79_O_8_P | 802.5513 | 11.97 | 7 | [M-H]^-^ | 2.63 | GL-RP-LC-QTOF-MS | ESI - | 3 | 0.75 | 4.84E-03ⱡ | 1.02 |  |
| PA 48:1 | C_51_H_99_O_8_P | 870.7078 | 25.86 | 8 | [M+Na]^+^ | 1.10 | GL-RP-LC-QTOF-MS | ESI + | 4 | 1.41 | 5.44E-03ⱡ | 1.04 |  |
| PA 48:2 | C_51_H_97_O_8_P | 868.6921 | 26.18 | 5 | [M+H]^+^ | 1.16 | GL-RP-LC-QTOF-MS | ESI + | 3 | 1.52 | 5.56E-03ⱡ | 1.13 |  |
| PA 50:0 | C_53_H_105_O_8_P | 900.7547 | 27.58 | 8 | [M+Na]^+^ | 3.95 | GL-RP-LC-QTOF-MS | ESI + | 3 | 1.57 | 1.26E-03ⱡ | 1.15 |  |
| PA O 32:2 | C_35_H_67_O_7_P | 630.4624 | 17.16 | 5 | [M+H]^+^ | 19.41 | GL-RP-LC-QTOF-MS | ESI + | 3 | 1.88 | 3.40E-03ⱡ | 1.40 |  |
| PA O 34:4 | C_37_H_67_O_7_P | 654.4624 | 16.64 | 4 | [M+H]^+^ | 16.05 | GL-RP-LC-QTOF-MS | ESI + | 3 | 1.45 | 1.25E-02ⱡ | 1.03 |  |
| PC P 34:1 // PC O 34:2 | C_42_H_82_NO_7_P | 743.5829 | 15.64 | 2 | [M+HCOOH-H]^-^ | 7.24 | GL-RP-LC-QTOF-MS | ESI +/- | 2 | 0.44 | 6.90E-05ⱡ | 1.72 |  |
| PC P 40:0 // PC O 40:1 | C_48_H_96_NO_7_P | 829.6924 | 19.19 | 8 | [M+HCOOH-H]^-^ | 9.09 | GL-RP-LC-QTOF-MS | ESI - | 3 | 0.53 | 3.08E-06ⱡ | 1.48 |  |
| PC 22:0 | C_46_H_92_NO_7_P | 801.6611 | 17.63 | 6 | [M+HCOOH-H]^-^ | 4.42 | GL-RP-LC-QTOF-MS | ESI - | 4 | 0.50 | 1.94E-06ⱡ | 1.53 |  |
| PC 32:2 | C_40_H_76_NO_8_P | 729.5309 | 12.48 | 1 | [M+H]^+^ | 1.38 | GL-RP-LC-QTOF-MS | ESI +/- | 2 | 0.50 | 3.47E-04ⱡ | 1.67 |  |
| PC 33:2 | C_41_H_78_NO_8_P | 743.5465 | 13.46 | 0 | [M+H]^+^ | 1.75 | GL-RP-LC-QTOF-MS | ESI +/- | 2 | 0.57 | 1.64E-05ⱡ | 1.42 |  |
| PC 34:3 | C_42_H_78_NO_8_P | 755.5465 | 13.28 | 1 | [M+H]^+^ | 2.04 | GL-RP-LC-QTOF-MS | ESI +/- | 2 | 0.72 | 9.82E-03ⱡ | 1.17 |  |
| PC P 34:5 // PC O 34:6 | C_42_H_76_NO_7_P | 737.5359 | 16.63 | 0 | [M-H]^-^ | 3.60 | GL-RP-LC-QTOF-MS | ESI - | 3 | 0.58 | 3.59E-03ⱡ | 1.35 |  |
| PC 35:2 | C_43_H_82_NO_8_P | 771.5778 | 15.36 | 5 | [M+H]^+^ | 2.89 | GL-RP-LC-QTOF-MS | ESI + | 3 | 0.66 | 2.56E-04ⱡ | 1.14 |  |
| PC P 36:1 // PC O 36:2 | C_44_H_86_NO_7_P | 771.6142 | 17.40 | 2 | [M+H]^+^ | 3.80 | GL-RP-LC-QTOF-MS | ESI +/- | 3 | 0.60 | 1.94E-06ⱡ | 1.38 |  |
| PC 36:3 | C_44_H_82_NO_8_P | 783.5778 | 15.14 | 5 | [M+H]^+^ | 3.07 | GL-RP-LC-QTOF-MS | ESI + | 2 | 0.65 | 5.36E-04ⱡ | 1.27 |  |
| PC P 36:2/0:0 // PC O 36:3/0:0 | C_44_H_84_NO_7_P | 769.5985 | 17.24 | 5 | [M+H]^+^ | 2.82 | GL-RP-LC-QTOF-MS | ESI + | 4 | 0.53 | 1.86E-05ⱡ | 1.46 |  |
| PC P 0:0/36:2 // PC O 0:0/36:3 | C_44_H_84_NO_7_P | 769.5985 | 16.14 | 4 | [M+H]^+^ | 3.95 | GL-RP-LC-QTOF-MS | ESI + | 3 | 0.64 | 1.70E-05ⱡ | 1.28 |  |
| PC 36:4 | C_44_H_80_NO_8_P | 781.5622 | 13.84 | 0 | [M+H]^+^ | 3.78 | GL-RP-LC-QTOF-MS | ESI +/- | 2 | 0.32 | 4.23E-05ⱡ | 2.10 |  |
| PC P 36:5 // PC O 36:6 | C_44_H_80_NO_7_P | 765.5672 | 14.68 | 0 | [M+HCOOH-H]^-^ | 13.04 | GL-RP-LC-QTOF-MS | ESI - | 2 | 0.53 | 1.64E-03ⱡ | 1.30 |  |
| PC 38:2 | C_46_H_88_NO_8_P | 813.6248 | 17.74 | 0 | [M+HCOOH-H]^-^ | 3.29 | GL-RP-LC-QTOF-MS | ESI - | 3 | 0.71 | 1.13E-02ⱡ | 1.06 |  |
| PC 38:3 | C_46_H_86_NO_8_P | 811.6091 | 16.92 | 5 | [M+H]^+^ | 4.22 | GL-RP-LC-QTOF-MS | ESI + | 2 | 0.69 | 3.03E-02ⱡ | 1.10 |  |
| PC 38:4 | C_46_H_84_NO_8_P | 809.5935 | 15.90 | 4 | [M+H]^+^ | 3.50 | GL-RP-LC-QTOF-MS | ESI + | 3 | 0.73 | 1.22E-02ⱡ | 1.07 |  |
| PC 38:6 | C_46_H_80_NO_8_P | 805.5622 | 13.92 | 0 | [M+HCOOH-H]^-^ | 2.05 | GL-RP-LC-QTOF-MS | ESI +/- | 2 | 0.50 | 1.87E-04ⱡ | 1.68 |  |
| PC P 40:4 // PC O 40:5 | C_48_H_90_NO_7_P | 823.6455 | 19.08 | 1 | [M+HCOOH-H]^-^ | 13.19 | GL-RP-LC-QTOF-MS | ESI - | 3 | 0.56 | 1.83E-04ⱡ | 1.37 |  |
| PC 41:4 | C_49_H_90_NO_8_P | 897.6459 | 18.38 | 5 | [M+HCOOH-H]^-^ | 8.01 | GL-RP-LC-QTOF-MS | ESI - | 3 | 0.46 | 3.61E-06ⱡ | 1.60 |  |
| PC P 0:0/42:3 // PC O 0:0/42:4 | C_50_H_92_NO_7_P | 849.6611 | 19.34 | 0 | [M+HCOOH-H]^-^ | 1.72 | GL-RP-LC-QTOF-MS | ESI - | 3 | 0.46 | 7.77E-05ⱡ | 1.61 |  |
| PC O 42:4 | C_50_H_94_NO_7_P | 851.6768 | 20.53 | 0 | [M+HCOOH-H]^-^ | 3.83 | GL-RP-LC-QTOF-MS | ESI - | 3 | 0.62 | 2.26E-03ⱡ | 1.19 |  |
| PC 43:4 | C_51_H_94_NO_8_P | 879.6720 | 19.87 | 6 | [M+HCOOH-H]^-^ | 2.24 | GL-RP-LC-QTOF-MS | ESI - | 3 | 0.51 | 9.12E-05ⱡ | 1.46 |  |
| PC 44:6 | C_52_H_94_NO_7_P | 875.6768 | 19.79 | 1 | [M+HCOOH-H]^-^ | 2.72 | GL-RP-LC-QTOF-MS | ESI - | 3 | 0.56 | 4.28E-04ⱡ | 1.29 |  |
| PC O 34:3 | C_42_H_80_NO_7_P | 741.5672 | 15.49 | 5 | [M+H]^+^ | 2.77 | GL-RP-LC-QTOF-MS | ESI + | 3 | 0.52 | 8.33E-05ⱡ | 1.57 |  |
| PC O 40:5 | C_48_H_88_NO_7_P | 821.6298 | 17.84 | 0 | [M+HCOOH-H]^-^ | 2.92 | GL-RP-LC-QTOF-MS | ESI - | 3 | 0.59 | 7.69E-05ⱡ | 1.29 |  |
| PC O 42:5 | C_50_H_92_NO_7_P | 849.6611 | 19.33 | 6 | [M+H]^+^ | 10.84 | GL-RP-LC-QTOF-MS | ESI + | 3 | 0.46 | 6.04E-06ⱡ | 1.52 |  |
| PC O 42:6 | C_50_H_90_NO_7_P | 847.6455 | 18.32 | 1 | [M+HCOOH-H]^-^ | 5.03 | GL-RP-LC-QTOF-MS | ESI - | 3 | 0.53 | 2.50E-05ⱡ | 1.51 |  |
| PC O 44:3 | C_52_H_100_NO_7_P | 881.7237 | 28.01 | 8 | [M+NH_4_]^+^ | 3.50 | GL-RP-LC-QTOF-MS | ESI + | 4 | 1.91 | 4.32E-03ⱡ | 1.31 |  |
| PC O 44:4 | C_52_H_98_NO_7_P | 879.7081 | 27.20 | 7 | [M+NH_4_]^+^ | 1.21 | GL-RP-LC-QTOF-MS | ESI + | 4 | 1.39 | 1.20E-03ⱡ | 1.04 |  |
| PC O 0:0/44:5 | C_52_H_96_NO_7_P | 877.6924 | 20.70 | 4 | [M+H]^+^ | 4.53 | GL-RP-LC-QTOF-MS | ESI + | 2 | 0.56 | 1.81E-03ⱡ | 1.33 |  |
| PE 25:4;O3 | C_30_H_52_NO_11_P | 633.3278 | 19.51 | 0 | [M-H]^-^ | 6.90 | GM-RP-LC-QTOF-MS | ESI - | 3 | 0.64 | 1.33E-04ⱡ | 1.26 |  |
| PE 26:1 | C_31_H_60_NO_8_P | 605.4057 | 20.19 | 6 | [M+NH_4_]^+^ | 5.40 | GL-RP-LC-QTOF-MS | ESI + | 3 | 0.28 | 3.00E-03ⱡ | 2.92 |  |
| PE 34:1 | C_39_H_76_NO_8_P | 717.5309 | 15.74 | 1 | [M-H]^-^ | 1.87 | GL-RP-LC-QTOF-MS | ESI - | 2 | 2.64 | 1.41E-02ⱡ | 1.56 |  |
| PE P 36:1 // PE O 36:2 | C_41_H_80_NO_7_P | 729.5672 | 18.45 | 0 | [M-H]^-^ | 5.90 | GL-RP-LC-QTOF-MS | ESI - | 3 | 0.52 | 3.60E-04ⱡ | 1.54 |  |
| PE P 36:3 // PE O 36:4 | C_41_H_76_NO_7_P | 725.5359 | 16.25 | 1 | [M-H]^-^ | 3.23 | GL-RP-LC-QTOF-MS | ESI - | 3 | 0.33 | 3.61E-06ⱡ | 2.07 |  |
| PE 36:3 | C_41_H_76_NO_8_P | 741.5309 | 13.26 | 1 | [M-H]^-^ | 2.88 | GL-RP-LC-QTOF-MS | ESI - | 2 | 0.76 | 1.16E-02ⱡ | 1.06 |  |
| PE P 36:2 // PE O 36:3 | C_41_H_78_NO_7_P | 727.5516 | 17.44 | 0 | [M-H]^-^ | 4.91 | GL-RP-LC-QTOF-MS | ESI - | 2 | 0.33 | 1.04E-04ⱡ | 2.06 |  |
| PE P 38:3 // PE O 38:4 | C_43_H_80_NO_7_P | 753.5672 | 18.05 | 2 | [M-H]^-^ | 4.15 | GL-RP-LC-QTOF-MS | ESI - | 3 | 0.54 | 8.20E-05ⱡ | 1.50 |  |
| PE 38:3 | C_44_H_82_NO_10_P | 815.5676 | 14.18 | 1 | [M-H]^-^ | 11.36 | GL-RP-LC-QTOF-MS | ESI - | 3 | 0.57 | 2.83E-04ⱡ | 1.44 |  |
| PC 35:4/0:0 | C_43_H_78_NO_8_P | 767.5465 | 16.27 | 0 | [M-H]^-^ | 5.85 | GL-RP-LC-QTOF-MS | ESI - | 2 | 0.55 | 2.81E-04ⱡ | 1.51 |  |
| PC 0:0/35:4 | C_43_H_78_NO_8_P | 767.5465 | 13.83 | 1 | [M-H]^-^ | 9.44 | GL-RP-LC-QTOF-MS | ESI - | 2 | 0.48 | 4.24E-04ⱡ | 1.63 |  |
| PE 40:6 iso 1 | C_45_H_80_NO_7_P | 777.5672 | 17.79 | 2 | [M-H]^-^ | 7.28 | GL-RP-LC-QTOF-MS | ESI - | 3 | 0.58 | 6.29E-05ⱡ | 1.38 |  |
| PE 40:6 iso 2 | C_45_H_80_NO_7_P | 777.5672 | 18.23 | 0 | [M-H]^-^ | 8.24 | GL-RP-LC-QTOF-MS | ESI - | 3 | 0.51 | 1.42E-03ⱡ | 1.51 |  |
| PE P 40:7 // PE O 40: 8 | C_45_H_76_NO_7_P | 773.5359 | 16.11 | 1 | [M-H]^-^ | 2.73 | GL-RP-LC-QTOF-MS | ESI - | 3 | 0.53 | 5.82E-05ⱡ | 1.51 |  |
| PE O 38:5 | C_43_H_78_NO_7_P | 751.5516 | 17.50 | 0 | [M-H]^-^ | 2.51 | GL-RP-LC-QTOF-MS | ESI - | 2 | 0.54 | 2.12E-03ⱡ | 1.48 |  |
| PE P 34:2 // PE O 34:3 | C_39_H_74_NO_7_P | 699.5203 | 15.68 | 1 | [M-H]^-^ | 3.91 | GL-RP-LC-QTOF-MS | ESI - | 2 | 0.38 | 2.43E-04ⱡ | 1.96 |  |
| PE P 36:4 // PE O 36:5 | C_41_H_74_NO_7_P | 723.5203 | 15.74 | 1 | [M-H]^-^ | 3.26 | GL-RP-LC-QTOF-MS | ESI - | 2 | 0.68 | 9.82E-03ⱡ | 1.15 |  |
| PE-NMe 42:8 | C_48_H_80_NO_8_P | 829.5622 | 13.86 | 1 | [M+HCOOH-H]^-^ | 4.33 | GL-RP-LC-QTOF-MS | ESI - | 3 | 0.56 | 2.66E-04ⱡ | 1.48 |  |
| PG 43:0 | C_49_H_97_O_10_P | 876.6819 | 18.33 | 6 | [M+Cl]^-^ | 10.44 | GL-RP-LC-QTOF-MS | ESI - | 4 | 0.63 | 3.33E-03ⱡ | 1.34 |  |
| PG 44:0 | C_50_H_99_O_10_P | 890.6976 | 19.12 | 3 | [M+Cl]^-^ | 13.14 | GL-RP-LC-QTOF-MS | ESI - | 3 | 0.66 | 2.10E-04ⱡ | 1.23 |  |
| PG O 32:0 | C_38_H_77_O_9_P | 708.5305 | 24.59 | 3 | [M+H]^+^ | 2.85 | GL-RP-LC-QTOF-MS | ESI + | 3 | 0.67 | 1.22E-02ⱡ | 1.27 |  |
| PG O 32:1 | C_38_H_75_O_9_P | 706.5149 | 18.22 | 3 | [M+HCOOH-H]^-^ | 10.13 | GL-RP-LC-QTOF-MS | ESI - | 3 | 1.52 | 6.87E-03ⱡ | 1.15 |  |
| PG O 32:2 | C_38_H_73_O_9_P | 704.4992 | 17.16 | 4 | [M+HCOOH-H]^-^ | 14.08 | GL-RP-LC-QTOF-MS | ESI - | 3 | 1.68 | 5.63E-03ⱡ | 1.24 |  |
| PG O 34:2 | C_40_H_77_O_9_P | 732.5305 | 18.82 | 4 | [M+HCOOH-H]^-^ | 10.52 | GL-RP-LC-QTOF-MS | ESI - | 3 | 1.42 | 1.04E-02ⱡ | 1.00 |  |
| PGP 36:1 | C_42_H_82_O_13_P_2_ | 856.5231 | 15.14 | 1 | [M+NH_4_]^+^ | 4.60 | GL-RP-LC-QTOF-MS | ESI + | 3 | 0.69 | 1.85E-04ⱡ | 1.17 |  |
| PI 36:3 | C_45_H_81_O_13_P | 860.5415 | 12.83 | 1 | [M-H]^-^ | 2.84 | GL-RP-LC-QTOF-MS | ESI - | 4 | 0.50 | 1.45E-03ⱡ | 1.50 |  |
| PI 38:3 | C_47_H_85_O_13_P | 888.5728 | 14.71 | 1 | [M-H]^-^ | 3.61 | GL-RP-LC-QTOF-MS | ESI - | 3 | 0.52 | 2.76E-04ⱡ | 1.48 |  |
| PI O 42:6 | C_51_H_89_O_12_P | 924.6092 | 12.30 | 4 | [M-H]^-^ | 6.93 | GL-RP-LC-QTOF-MS | ESI - | 4 | 0.46 | 1.12E-03ⱡ | 1.50 |  |
| PIM1 36:0 | C_51_H_97_O_18_P | 1028.6413 | 20.18 | 4 | [M+HCOOH-H]^-^ | 13.51 | GM-RP-LC-QTOF-MS | ESI - | 4 | 0.49 | 1.33E-04ⱡ | 1.57 |  |
| LPS O 21:0;O | C_27_H_56_NO_9_P | 569.3693 | 26.24 | 3 | [M-H]^-^ | 5.59 | GM-RP-LC-QTOF-MS | ESI - | 3 | 0.74 | 1.57E-02ⱡ | 1.16 |  |
| LPS 20:4 | C_26_H_44_NO_9_P | 545.2754 | 20.00 | 5 | [M-H]^-^ | 9.12 | GM-RP-LC-QTOF-MS | ESI - | 3 | 0.69 | 3.97E-02ⱡ | 1.14 |  |
| PS 36:3 | C_42_H_76_NO_10_P | 785.5207 | 15.75 | 4 | [M-H]^-^ | 5.52 | GL-RP-LC-QTOF-MS | ESI - | 3 | 1.98 | 7.01E-03ⱡ | 1.32 |  |
| PS 36:4 | C_42_H_74_NO_10_P | 783.50500 | 14.63 | 3 | [M-H]^-^ | 2.24 | GL-RP-LC-QTOF-MS | ESI - | 3 | 1.66 | 2.38E-02ⱡ | 1.20 |  |
| PE-NMe2 36:7 | C_43_H_72_NO_8_P | 761.5000 | 14.71 | 3 | [M+HCOOH-H]^-^ | 4.36 | GL-RP-LC-QTOF-MS | ESI - | 3 | 1.78 | 3.56E-03ⱡ | - |  |
| PS 39:0 | C_45_H_88_NO_10_P | 833.6146 | 17.25 | 0 | [M-H-H_2_O]^-^ | 8.65 | GL-RP-LC-QTOF-MS | ESI - | 3 | 0.49 | 5.27E-05ⱡ | 1.52 |  |
| PS 39:5 | C_45_H_78_NO_10_P | 823.5363 | 13.25 | 3 | [M+HCOOH-H]^-^ | 2.32 | GL-RP-LC-QTOF-MS | ESI - | 3 | 0.73 | 6.65E-03ⱡ | 1.13 |  |
| PS 40:2 | C_46_H_86_NO_10_P | 843.5989 | 15.89 | 0 | [M-H]^-^ | 2.78 | GL-RP-LC-QTOF-MS | ESI - | 3 | 0.59 | 2.66E-04ⱡ | 1.31 |  |
| PS 40:6 | C_46_H_78_NO_9_P | 819.5414 | 17.50 | 3 | [M-H]^-^ | 3.06 | GL-RP-LC-QTOF-MS | ESI - | 3 | 0.64 | 3.66E-04ⱡ | 1.20 |  |
| PS 41:6 | C_47_H_80_NO_10_P | 849.5520 | 13.85 | 2 | [M+Na]^+^ | 2.15 | GL-RP-LC-QTOF-MS | ESI +/- | 3 | 0.38 | 2.40E-08ⱡ | 1.98 |  |
| PS 44:1 | C_50_H_96_NO_10_P | 901.6772 | 26.42 | 9 | [M+NH_4_]^+^ | 3.61 | GL-RP-LC-QTOF-MS | ESI + | 3 | 1.90 | 4.04E-04ⱡ | 1.32 |  |
| PS 44:3 | C_50_H_92_NO_10_P | 897.6459 | 25.36 | 9 | [M+NH_4_]^+^ | 1.60 | GL-RP-LC-QTOF-MS | ESI + | 4 | 1.72 | 2.02E-02ⱡ | 1.13 |  |
| PS 46:0 | C_52_H_102_NO_10_P | 931.7241 | 27.93 | 9 | [M+NH_4_]^+^ | 3.84 | GL-RP-LC-QTOF-MS | ESI + | 4 | 2.10 | 1.51E-05ⱡ | 1.56 |  |
| PS 46:1 | C_52_H_100_NO_10_P | 929.7085 | 27.51 | 9 | [M+NH_4_]^+^ | 2.94 | GL-RP-LC-QTOF-MS | ESI + | 4 | 2.10 | 1.51E-05ⱡ | 1.56 |  |
| PS 46:2 iso 1 | C_52_H_98_NO_10_P | 927.6928 | 26.58 | 9 | [M+NH_4_]^+^ | 5.60 | GL-RP-LC-QTOF-MS | ESI + | 4 | 1.38 | 8.82E-03ⱡ | 1.03 |  |
| PS 46:2 iso 2 | C_52_H_98_NO_10_P | 927.6928 | 26.76 | 9 | [M+NH_4_]^+^ | 4.52 | GL-RP-LC-QTOF-MS | ESI + | 4 | 1.38 | 8.82E-03ⱡ | 1.03 |  |
| PS 46:3 | C_52_H_96_NO_10_P | 925.6772 | 26.11 | 9 | [M+NH_4_]^+^ | 2.52 | GL-RP-LC-QTOF-MS | ESI + | 4 | 1.38 | 8.82E-03ⱡ | 1.03 |  |
| PS 46:4 | C_52_H_94_NO_10_P | 923.6615 | 25.66 | 8 | [M+NH_4_]^+^ | 1.71 | GL-RP-LC-QTOF-MS | ESI + | 4 | 1.58 | 8.82E-03ⱡ | 1.03 |  |
| PS O 35:3 | C_41_H_76_NO_9_P | 757.5258 | 14.71 | 0 | [M-H-H_2_O]^-^ | 2.15 | GL-RP-LC-QTOF-MS | ESI - | 3 | 1.88 | 1.40E-02ⱡ | 1.25 |  |
| PS O 38:7 | C_44_H_74_NO_9_P | 791.5101 | 15.75 | 3 | [M-H]^-^ | 4.95 | GL-RP-LC-QTOF-MS | ESI - | 3 | 0.74 | 6.40E-03ⱡ | - |  |
| PS O 39:2 | C_45_H_86_NO_9_P | 815.6040 | 16.13 | 1 | [M-H]^-^ | 2.78 | GL-RP-LC-QTOF-MS | ESI - | 3 | 0.56 | 3.61E-06ⱡ | 1.46 |  |
| PS 24:2;O2 | C_30_H_54_NO_12_P | 651.3384 | 20.19 | 2 | [M-H-H_2_O]^-^ | 6.90 | GM-RP-LC-QTOF-MS | ESI - | 3 | 0.69 | 6.21E-05ⱡ | 1.17 |  |
| ***Sphingolipids*** | | | | | | | | | | | | |  |
| Cer 32:1;O2 | C_32_H_63_NO_3_ | 509.4808 | 12.30 | 1 | [M+Cl]^-^ | 7.82 | GL-RP-LC-QTOF-MS | ESI - | 3 | 0.72 | 9.76E-03ⱡ | 1.24 |  |
| Cer 34:0;O2 | C_34_H_69_NO_3_ | 539.5277 | 14.92 | 1 | [M-H]^-^ | 3.75 | GL-RP-LC-QTOF-MS | ESI - | 3 | 2.22 | 2.74E-02ⱡ | 1.19 |  |
| Cer 34:1;O2 | C_34_H_67_NO_3_ | 537.5121 | 14.27 | 1 | [M-H]^-^ | 2.91 | GL-RP-LC-QTOF-MS | ESI +/- | 2 | 2.05 | 6.84E-03ⱡ | 1.23 |  |
| Cer 36:0;O2 | C_36_H_73_NO_3_ | 567.5590 | 16.76 | 1 | [M-H]^-^ | 11.44 | GL-RP-LC-QTOF-MS | ESI - | 3 | 3.12 | 8.37E-03ⱡ | 1.49 |  |
| Cer 36:1;O2 | C_36_H_71_NO_3_ | 565.5434 | 16.16 | 1 | [M-H]^-^ | 4.06 | GL-RP-LC-QTOF-MS | ESI - | 3 | 2.71 | 3.63E-04ⱡ | 1.69 |  |
| Cer 36:1;O3 | C_36_H_71_NO_4_ | 581.5383 | 14.27 | 1 | [M+HCOOH-H]^-^ | 2.59 | GL-RP-LC-QTOF-MS | ESI - | 3 | 1.85 | 5.63E-03ⱡ | 1.22 |  |
| Cer 38:1;O2 | C_38_H_75_NO_3_ | 593.5747 | 17.94 | 0 | [M-H]^-^ | 3.56 | GL-RP-LC-QTOF-MS | ESI - | 3 | 1.66 | 3.55E-02ⱡ | 1.16 |  |
| Cer 39:1;O2 | C_39_H_77_NO_3_ | 607.5903 | 18.93 | 1 | [M-H]^-^ | 4.31 | GL-RP-LC-QTOF-MS | ESI - | 3 | 0.69 | 1.49E-02ⱡ | 1.20 |  |
| Cer 40:0;O3 | C_40_H_81_NO_4_ | 639.6166 | 18.73 | 1 | M+HCOOH-H | 1.63 | GL-RP-LC-QTOF-MS | ESI - | 3 | 0.72 | 2.28E-02ⱡ | 1.09 |  |
| Cer 41:0;O2 | C_41_H_83_NO_3_ | 637.6373 | 20.68 | 1 | [M+Cl]^-^ | 5.98 | GL-RP-LC-QTOF-MS | ESI - | 3 | 0.61 | 5.74E-03ⱡ | 1.36 |  |
| Cer 41:1;O2 | C_41_H_81_NO_3_ | 635.6216 | 20.27 | 0 | [M-H]^-^ | 4.85 | GL-RP-LC-QTOF-MS | ESI - | 2 | 0.72 | 1.03E-02ⱡ | 1.12 |  |
| Cer 41:2;O2 | C_41_H_79_NO_3_ | 633.6060 | 19.41 | 0 | [M-H]^-^ | 3.90 | GL-RP-LC-QTOF-MS | ESI - | 3 | 0.69 | 1.44E-02ⱡ | 1.26 |  |
| Cer 42:0;O2 | C_42_H_85_NO_3_ | 651.6529 | 21.34 | 0 | [M-H]^-^ | 9.93 | GL-RP-LC-QTOF-MS | ESI - | 3 | 0.64 | 3.60E-02ⱡ | 1.21 |  |
| Cer 42:1;O2 | C_42_H_83_NO_3_ | 649.6373 | 20.96 | 0 | [M-H]^-^ | 1.75 | GL-RP-LC-QTOF-MS | ESI - | 2 | 0.78 | 4.46E-02ⱡ | - |  |
| Cer 42:2;O | C_42_H_81_NO_2_ | 631.6267 | 20.22 | 1 | [M+HCOOH-H]^-^ | 4.64 | GL-RP-LC-QTOF-MS | ESI - | 3 | 2.34 | 3.05E-02ⱡ | 1.29 |  |
| Cer 42:2;O2 | C_42_H_81_NO_3_ | 647.6216 | 19.80 | 0 | [M-H]^-^ | 4.05 | GL-RP-LC-QTOF-MS | ESI +/- | 2 | 1.71 | 2.62E-02ⱡ | 1.22 |  |
| Cer 43:1;O2 | C_43_H_85_NO_3_ | 663.6529 | 21.43 | 1 | [M-H]^-^ | 5.40 | GL-RP-LC-QTOF-MS | ESI - | 3 | 0.61 | 1.64E-03ⱡ | 1.33 |  |
| Cer 44:1;O2 | C_44_H_87_NO_3_ | 677.6686 | 22.09 | 0 | [M+Cl]^-^ | 5.90 | GL-RP-LC-QTOF-MS | ESI - | 3 | 0.72 | 1.09E-02ⱡ | 1.03 |  |
| CerPE 34:1;O2 | C_36_H_73_N_2_O_1_P | 660.5206 | 10.97 | 0 | [M-H]^-^ | 4.35 | GL-RP-LC-QTOF-MS | ESI - | 3 | 0.55 | 1.16E-04ⱡ | 1.54 |  |
| CerPE 34:2;O3 | C_36_H_71_N_2_O_7_P | 674.4999 | 18.19 | 9 | [M-H]^-^ | 11.09 | GL-RP-LC-QTOF-MS | ESI - | 3 | 1.63 | 3.66E-04ⱡ | 1.28 |  |
| HexCer 32:2;O2 | C_38_H_71_NO_8_ | 669.5180 | 14.23 | 3 | [M-H-H_2_O]^-^ | 4.51 | GL-RP-LC-QTOF-MS | ESI - | 3 | 1.80 | 5.54E-04ⱡ | 1.24 |  |
| HexCer 32:3;O2 iso 1 | C_38_H_69_NO_8_ | 667.5023 | 24.86 | 1 | [M+NH_4_]^+^ | 3.15 | GL-RP-LC-QTOF-MS | ESI + | 3 | 0.56 | 4.04E-04ⱡ | 1.47 |  |
| HexCer 32:3;O2 iso 2 | C_38_H_69_NO_8_ | 667.5023 | 14.20 | 5 | [M-H-H_2_O]^-^ | 4.34 | GL-RP-LC-QTOF-MS | ESI - | 3 | 2.34 | 6.36E-04ⱡ | 1.40 |  |
| Hex2Cer 34:1;O2 | C_46_H_87_NO_13_ | 861.6177 | 12.34 | 1 | [M-H]^-^ | 2.56 | GL-RP-LC-QTOF-MS | ESI - | 3 | 0.51 | 5.08E-04ⱡ | 1.54 |  |
| HexCer 34:2;O2 | C_40_H_75_NO_8_ | 697.5493 | 16.14 | 4 | [M-H-H_2_O]^-^ | 6.37 | GL-RP-LC-QTOF-MS | ESI - | 4 | 2.72 | 7.77E-05ⱡ | 1.77 |  |
| HexCer 38:1;O2 | C_44_H_85_NO_8_ | 755.6275 | 16.69 | 0 | [M+HCOOH-H]^-^ | 6.18 | GL-RP-LC-QTOF-MS | ESI - | 3 | 0.69 | 4.13E-03ⱡ | 1.08 |  |
| HexCer 39:1;O2 | C_45_H_87_NO_8_ | 769.6432 | 17.66 | 1 | [M+HCOOH-H]^-^ | 10.40 | GL-RP-LC-QTOF-MS | ESI - | 3 | 0.27 | 1.64E-05ⱡ | 2.16 |  |
| HexCer 39:2;O2 | C_45_H_85_NO_8_ | 767.6275 | 20.27 | 2 | [M-H-H_2_O]^-^ | 4.80 | GL-RP-LC-QTOF-MS | ESI - | 4 | 0.72 | 1.03E-02ⱡ | 1.09 |  |
| HexCer 40:1;O2 iso 1 | C_46_H_89_NO_8_ | 783.6588 | 21.34 | 2 | [M-H-H_2_O]^-^ | 8.78 | GL-RP-LC-QTOF-MS | ESI - | 4 | 0.63 | 4.01E-03ⱡ | 1.29 |  |
| HexCer 40:1;O2 iso 2 | C_46_H_89_NO_8_ | 783.6588 | 18.37 | 1 | [M-H]^-^ | 1.95 | GL-RP-LC-QTOF-MS | ESI - | 3 | 0.44 | 3.41E-06ⱡ | 1.67 |  |
| HexCer 40:2;O2 iso 1 | C_46_H_87_NO_8_ | 781.6432 | 17.40 | 0 | [M+HCOOH-H]^-^ | 3.15 | GL-RP-LC-QTOF-MS | ESI - | 3 | 0.53 | 1.58E-04ⱡ | 1.49 |  |
| HexCer 40:2;O2 iso 2 | C_46_H_87_NO_8_ | 781.6432 | 20.96 | 3 | [M-H-H_2_O]^-^ | 3.71 | GL-RP-LC-QTOF-MS | ESI - | 3 | 0.76 | 8.17E-03ⱡ | - |  |
| HexCer 41:1;O2 | C_47_H_91_NO_8_ | 797.6745 | 19.13 | 0 | [M-H]^-^ | 4.10 | GL-RP-LC-QTOF-MS | ESI - | 3 | 0.42 | 1.94E-06ⱡ | 1.75 |  |
| HexCer 41:2;O2 iso 1 | C_47_H_89_NO_8_ | 795.6588 | 18.23 | 1 | [M+HCOOH-H]^-^ | 4.06 | GL-RP-LC-QTOF-MS | ESI - | 3 | 0.41 | 1.41E-06ⱡ | 1.78 |  |
| HexCer 41:2;O2 iso 2 | C_47_H_89_NO_8_ | 795.6588 | 21.44 | 3 | [M-H-H_2_O]^-^ | 8.39 | GL-RP-LC-QTOF-MS | ESI - | 3 | 0.62 | 4.59E-04ⱡ | 1.29 |  |
| HexCer 42:1;O2 | C_48_H_93_NO_8_ | 811.6901 | 19.87 | 0 | [M-H]^-^ | 2.55 | GL-RP-LC-QTOF-MS | ESI - | 3 | 0.55 | 1.15E-04ⱡ | 1.37 |  |
| HexCer 42:1;O3 | C_48_H_93_NO_9_ | 827.6850 | 19.39 | 4 | [M-H]^-^ | 1.53 | GL-RP-LC-QTOF-MS | ESI - | 3 | 0.68 | 2.12E-04ⱡ | 1.12 |  |
| HexCer 42:2;O | C_48_H_91_NO_8_ | 809.6745 | 19.01 | 0 | [M+HCOOH-H]^-^ | 2.26 | GL-RP-LC-QTOF-MS | ESI - | 3 | 0.56 | 6.29E-05ⱡ | 1.40 |  |
| Hex2Cer 42:2;O2 | C_54_H_101_NO_13_ | 971.7273 | 18.07 | 1 | [M+HCOOH-H]^-^ | 2.38 | GL-RP-LC-QTOF-MS | ESI - | 4 | 0.59 | 7.28E-05ⱡ | 1.28 |  |
| SHexCer 34:1;O2 | C_40_H_77_NO_11_S | 779.5217 | 10.61 | 0 | [M-H]^-^ | 3.04 | GL-RP-LC-QTOF-MS | ESI - | 3 | 0.47 | 5.08E-04ⱡ | 1.64 |  |
| SHexCer 34:1;O3 | C_40_H_77_NO_12_S | 795.5167 | 10.10 | 1 | [M-H]^-^ | 1.23 | GL-RP-LC-QTOF-MS | ESI - | 3 | 0.43 | 5.36E-05ⱡ | 1.78 |  |
| SM 32:1;O2 iso 1 | C_37_H_75_N_2_O_6_P | 674.5363 | 10.97 | 5 | [M+H]^+^ | 4.28 | GL-RP-LC-QTOF-MS | ESI +/- | 2 | 0.55 | 8.75E-05ⱡ | 1.43 |  |
| SM 32:1;O2 iso 2 | C_37_H_75_N_2_O_6_P | 674.5363 | 11.96 | 0 | [M-H]^-^ | 3.87 | GL-RP-LC-QTOF-MS | ESI - | 3 | 0.74 | 4.84E-03ⱡ | 1.08 |  |
| SM 37:1;O2 | C_42_H_85_N_2_O_6_P | 744.6145 | 15.91 | 1 | [M+HCOOH-H]^-^ | 5.71 | GL-RP-LC-QTOF-MS | ESI - | 3 | 0.68 | 7.45E-05ⱡ | 1.14 |  |
| SM 38:1;O2 | C_43_H_87_N_2_O_6_P | 758.6302 | 17.00 | 5 | [M+H]^+^ | 4.90 | GL-RP-LC-QTOF-MS | ESI +/- | 2 | 0.40 | 6.22E-07ⱡ | 1.82 |  |
| SM 39:1;O2 | C_44_H_89_N_2_O_6_P | 772.6458 | 17.88 | 1 | [M+H]^+^ | 1.93 | GL-RP-LC-QTOF-MS | ESI +/- | 3 | 0.39 | 2.78E-08ⱡ | 1.90 |  |
| SM 39:2;O2 | C_44_H_87_N_2_O_6_P | 770.6302 | 16.61 | 1 | [M+HCOOH-H]^-^ | 4.92 | GL-RP-LC-QTOF-MS | ESI - | 3 | 0.60 | 4.24E-04ⱡ | 1.40 |  |
| SM 40:0;O2 | C_45_H_93_N_2_O_6_P | 788.6771 | 19.03 | 2 | [M+HCOOH-H]^-^ | 5.76 | GL-RP-LC-QTOF-MS | ESI - | 3 | 0.56 | 6.06E-05ⱡ | 1.34 |  |
| SM 40:1;O2 | C_45_H_91_N_2_O_6_P | 786.6615 | 18.44 | 5 | [M+H]^+^ | 3.21 | GL-RP-LC-QTOF-MS | ESI +/- | 2 | 0.68 | 1.10E-04ⱡ | 1.10 |  |
| SM 40:2;O2 iso 1 | C_45_H_89_N_2_O_6_P | 784.6458 | 17.24 | 5 | [M+H]^+^ | 5.24 | GL-RP-LC-QTOF-MS | ESI + | 2 | 0.71 | 9.27E-03ⱡ | - |  |
| SM 40:2;O2 iso 2 | C_45_H_89_N_2_O_6_P | 784.6458 | 17.47 | 5 | [M+H]^+^ | 4.36 | GL-RP-LC-QTOF-MS | ESI + | 2 | 0.70 | 1.41E-03ⱡ | 1.15 |  |
| SM 41:1;O2 | C_46_H_93_N_2_O_6_P | 800.6771 | 19.23 | 5 | [M+H]^+^ | 4.88 | GL-RP-LC-QTOF-MS | ESI +/- | 2 | 0.54 | 5.10E-07ⱡ | 1.49 |  |
| SM 41:2;O2 | C_46_H_91_N_2_O_6_P | 798.6615 | 18.33 | 1 | [M+HCOOH-H]^-^ | 4.52 | GL-RP-LC-QTOF-MS | ESI +/- | 2 | 0.58 | 5.82E-05ⱡ | 1.47 |  |
| SM 42:1;O2 | C_47_H_95_N_2_O_6_P | 814.6928 | 19.98 | 5 | [M+H]^+^ | 3.86 | GL-RP-LC-QTOF-MS | ESI +/- | 2 | 0.65 | 4.96E-05ⱡ | 1.22 |  |
| SM 42:2;O2 | C_47_H_93_N_2_O_6_P | 812.6771 | 19.11 | 5 | [M+H]^+^ | 16.23 | GL-RP-LC-QTOF-MS | ESI +/- | 2 | 0.62 | 9.87E-05ⱡ | 1.35 |  |
| SM 43:1;O2 | C_48_H_97_N_2_O_6_P | 828.7084 | 20.47 | 0 | [M+HCOOH-H]^-^ | 3.29 | GL-RP-LC-QTOF-MS | ESI - | 3 | 0.58 | 1.66E-04ⱡ | 1.41 |  |
| SM 43:2;O2 | C_48_H_95_N_2_O_6_P | 826.6928 | 19.87 | 0 | [M+HCOOH-H]^-^ | 4.22 | GL-RP-LC-QTOF-MS | ESI - | 3 | 0.64 | 6.80E-04ⱡ | 1.25 |  |
| NeuAcHexCer 34:1;O2 | C_51_H_94_N_2_O_16_ | 990.6603 | 21.71 | 9 | [M+H]^+^ | 3.04 | GM-RP-LC-QTOF-MS | ESI +/- | 4 | 0.71 | 1.68E-02 | 1.93 |  |
| Hex(2)-HexNAc-Cer 36:1;O2 | C_56_H_104_N_2_O_18_ | 1092.7284 | 26.23 | 2 | [M-H]^-^ | 9.93 | GM-RP-LC-QTOF-MS | ESI - | 4 | 0.65 | 1.41E-02ⱡ | 1.56 |  |
| ***Steroids*** | | | | | | | | | | | | |  |
| Androstenol | C_19_H_30_O | 274.2297 | 25.53 | 5 | [M-H-H_2_O]^-^ | 5.09 | GL-RP-LC-QTOF-MS | ESI + | 3 | 0.55 | 3.05E-07ⱡ | 1.47 |  |
| CE 18:1 | C_45_H_78_O_2_ | 650.6002 | 26.27 | 5 | [M+NH_4_]^+^ | 1.50 | GL-RP-LC-QTOF-MS | ESI + | 4 | 0.69 | 7.99E-03ⱡ | 1.19 |  |
| CE 18:2 | C_45_H_76_O_2_ | 648.5845 | 25.53 | 5 | [M+H]^+^ | 1.89 | GL-RP-LC-QTOF-MS | ESI + | 3 | 0.62 | 6.72E-07ⱡ | 1.31 |  |
| CE 18:3 | C_45_H_74_O_2_ | 646.5689 | 24.86 | 5 | [M+NH_4_]^+^ | 1.98 | GL-RP-LC-QTOF-MS | ESI + | 3 | 0.50 | 2.88E-04ⱡ | 1.64 |  |
| CE 20:0 | C_47_H_84_O_2_ | 680.6471 | 21.44 | 7 | [M+HCOOH-H]^-^ | 8.18 | GL-RP-LC-QTOF-MS | ESI - | 3 | 0.63 | 1.18E-03ⱡ | 1.28 |  |
| CE 20:1 | C_47_H_82_O_2_ | 678.6315 | 20.85 | 7 | [M+HCOOH-H]^-^ | 2.47 | GL-RP-LC-QTOF-MS | ESI - | 3 | 0.75 | 2.24E-02ⱡ | 1.07 |  |
| CE 20:3 | C_47_H_78_O_2_ | 674.6002 | 25.84 | 5 | [M+NH_4_]^+^ | 6.59 | GL-RP-LC-QTOF-MS | ESI + | 3 | 0.57 | 2.43E-03ⱡ | 1.47 |  |
| CE 20:4 | C_47_H_76_O_2_ | 672.5845 | 26.27 | 2 | [M+H]^+^ | 2.33 | GL-RP-LC-QTOF-MS | ESI + | 3 | 0.71 | 8.21E-04ⱡ | 1.12 |  |
| CE 20:5 | C_47_H_74_O_2_ | 670.5689 | 24.59 | 5 | [M+Na]^+^ | 4.63 | GL-RP-LC-QTOF-MS | ESI + | 3 | 0.65 | 1.16E-02ⱡ | 1.29 |  |
| CE 22:6 | C_49_H_76_O_2_ | 696.5845 | 25.83 | 1 | [M+H]^+^ | 7.94 | GL-RP-LC-QTOF-MS | ESI + | 3 | 0.53 | 6.67E-04ⱡ | 1.63 |  |
| Testosterone sulfate | C_19_H_28_O_5_S | 368.1657 | 8.19 | 0 | [M-H]^-^ | 5.35 | GM-RP-LC-QTOF-MS | ESI - | 3 | 0.42 | 6.44E-04ⱡ | 2.25 |  |
| Hydroxy DHEA sulfate | C_19_H_28_O_6_S | 384.1607 | 4.22 | 2 | [M-H]^-^ | 7.18 | GM-RP-LC-QTOF-MS | ESI - | 3 | 0.67 | 7.91E-02ⱡ | 1.17 |  |
| Pregnenolone sulfate | C_21_H_32_O_5_S | 396.1970 | 11.31 | 2 | [M-H]^-^ | 9.51 | GM-RP-LC-QTOF-MS | ESI - | 3 | 0.50 | 7.75E-03ⱡ | 1.47 |  |
| ***Sterol Lipids*** | | | | | | | | | | | | |  |
| Castasterone | C_28_H_48_O_5_ | 464.3502 | 33.62 | 0 | [M-H]^-^ | 5.55 | GM-RP-LC-QTOF-MS | ESI - | 3 | 0.55 | 4.10E-04ⱡ | 1.30 |  |
| Dihydrotestosterone sulfate | C_19_H_30_O_5_S | 370.1814 | 9.50 | 3 | [M-H]^-^ | 5.72 | GM-RP-LC-QTOF-MS | ESI - | 3 | 0.47 | 6.83E-03ⱡ | 2.21 |  |
| 20:3-Glc-cholesterol | C_53_H_88_O_7_ | 836.6530 | 17.02 | 8 | [M+Cl]^-^ | 2.75 | GL-RP-LC-QTOF-MS | ESI - | 4 | 0.39 | 2.78E-08ⱡ | 1.89 |  |
| Cholesterol | C_27_H_46_O | 386.3549 | 24.59 | 5 | [M+H-H_2_O]^+^ | 5.41 | GL-RP-LC-QTOF-MS | ESI + | 2 | 0.66 | 1.24E-02ⱡ | 1.40 |  |
| Cholesteryl nitrolinoleate | C_45_H_75_NO_4_ | 693.5696 | 19.78 | 9 | [M+Cl]^-^ | 16.26 | GL-RP-LC-QTOF-MS | ESI - | 4 | 2.00 | 5.23E-04ⱡ | 1.45 |  |
| Ecdysone palmitate | C_43_H_74_O_7_ | 702.5435 | 18.20 | 4 | [M-H-H_2_O]^-^ | 15.22 | GL-RP-LC-QTOF-MS | ESI - | 3 | 1.70 | 7.92E-03ⱡ | 1.28 |  |
| Sitostanyl oleate | C_45_H_82_O_2_ | 654.6315 | 20.68 | 8 | [M+HCOOH-H]^-^ | 11.47 | GL-RP-LC-QTOF-MS | ESI - | 3 | 0.63 | 7.25E-03ⱡ | 1.32 |  |
| DHEA | C_19_H_28_O_2_ | 288.2089 | 8.10 | 3 | [M+H-H_2_O]^+^ | 5.11 | GM-RP-LC-QTOF-MS | ESI + | 3 | 0.43 | 1.00E-03ⱡ | 2.43 |  |
| Campesteryl glucoside | C_34_H_58_O_6_ | 562.4233 | 34.79 | 1 | [M+H]^+^ | 17.62 | GM-RP-LC-QTOF-MS | ESI + | 3 | 0.49 | 1.00E-03ⱡ | 1.70 |  |
| ***Fatty Acyls*** | | | | | | | | | | | | |  |
| Didecanoyl-docosane-2,3-diol | C_42_H_82_O_4_ | 650.6213 | 19.79 | 4 | [M+Cl]^-^ | 3.80 | GL-RP-LC-QTOF-MS | ESI - | 4 | 1.50 | 1.47E-02ⱡ | 1.09 |  |
| FA 38:5 | C_38_H_66_O_2_ | 554.5063 | 14.21 | 8 | [M+HCOOH-H]^-^ | 7.02 | GL-RP-LC-QTOF-MS | ESI - | 3 | 2.14 | 3.10E-04ⱡ | 1.43 |  |
| Hepten-2,5-dione | C_7_H_10_O_2_ | 126.0681 | 0.69 | 1 | [M-H-H_2_O]^-^ | 4.98 | GL-RP-LC-QTOF-MS | ESI - | 3 | 0.60 | 2.58E-03ⱡ | 1.98 |  |
| Oleic acid | C_18_H_34_O_2_ | 282.2559 | 17.80 | 2 | [M-H]^-^ | 19.81 | GL-RP-LC-QTOF-MS | ESI - | 3 | 1.44 | 3.59E-02ⱡ | - |  |
| Arachidonic acid | C_20_H_32_O_2_ | 304.2402 | 31.42 | 5 | [M+H]^+^ | 6.10 | GM-RP-LC-QTOF-MS | ESI + | 3 | 1.37 | 3.2E-02ⱡ | 1.20 |  |
| 2-Oxo-hexenoic acid | C_6_H_8_O_3_ | 128.0473 | 1.61 | 8 | [M-H-H_2_O]^-^ | 7.76 | GM-RP-LC-QTOF-MS | ESI - | 3 | 0.26 | 1.96E-04ⱡ | 1.75 |  |
| 13-Oxo-tridecadienoic acid | C_13_H_20_O_3_ | 224.1412 | 14.71 | 2 | [M-H]^-^ | 5.37 | GM-RP-LC-QTOF-MS | ESI - | 3 | 0.48 | 6.64E-05ⱡ | 1.70 |  |
| Nisinic acid | C_24_H_36_O_2_ | 374.2821 | 18.37 | 4 | [M+H] | 9.51 | GM-RP-LC-QTOF-MS | ESI + | 3 | 0.51 | 3.60E-02 | 1.60 |  |
| Linoleic acid | C_18_H_32_O_2_ | 280.2402 | 19.51 | 1 | [M-H]^-^ | 8.98 | GM-RP-LC-QTOF-MS | ESI - | 2 | 0.60 | 1.33E-04ⱡ | 1.35 |  |
| Methyl-hydroxy-eicosanoate | C_20_H_40_O_3_ | 328.2977 | 35.76 | 2 | [M-H]^-^ | 10.70 | GM-RP-LC-QTOF-MS | ESI - | 3 | 0.41 | 7.88E-03ⱡ | 1.02 |  |
| Myristic acid | C_14_H_28_O_2_ | 117.0366* | 16.72 | - | - | 4.37 | GC-QTOF-MS | - | 1 | 0.63 | 4.26E-02 ⱡ | 1.13 |  |
| 2-Hydroxy-3-methylbutyric acid | C_5_H_10_O_3_ | 145.1039* | 8.35 | - | - | 8.27 | GC-QTOF-MS | - | 1 | 2.96 | 4.12E-02 ⱡ | 1.35 |  |
| Caprylic acid | C_8_H_16_O_2_ | 201.1301* | 9.74 | - | - | 5.11 | GC-QTOF-MS | - | 1 | 0.79 | 2.79E-02 ⱡ | - |  |
| Lauric acid | C_12_H_24_O_2_ | 117.0366* | 14.60 | - | - | 8.01 | GC-QTOF-MS | - | 1 | 0.45 | 4.56E-02 ⱡ | - |  |
| ***Amino acids*** | | | | | | | | | | | | |  |
| Leucylproline | C_11_H_20_N_2_O_3_ | 228.1474 | 1.12 | 3 | [M+H]^+^ | 3.64 | GM-RP-LC-QTOF-MS | ESI + | 3 | 2.13 | 3.00E-03ⱡ | 1.61 |  |
| 4-Acetamido-aminobutanoic acid | C_6_H_12_N_2_O_3_ | 160.0848 | 1.18 | 2 | [M+H-H_2_O]^+^ | 5.92 | GM-RP-LC-QTOF-MS | ESI + | 3 | 3.02 | - | 1.47 |  |
| Cystine | C_6_H_12_N_2_O_4_S_2_ | 148.0618 | 21.11 | - | - | 7.77 | GC-QTOF-MS | - | 1 | 0.62 | 4.12E-02 ⱡ | 1.24 |  |
| Tyrosine | C_9_H_11_NO_3_ | 218.1027 | 17.81 | - | - | 12.51 | GC-QTOF-MS | - | 1 | 0.83 | - | 1.07 |  |
| Glutamic acid | C_5_H_9_NO_4_ | 246.1326 | 14.34 | - | - | 3.72 | GC-QTOF-MS | - | 1 | 3.94 | 2.40E-02 | 1.28 |  |
| Aminobutanoic acid | C_4_H_9_NO_2_ | 58.0651 | 6.26 | - | - | 26.24 | GC-QTOF-MS | - | 1 | 2.95 | 1.35E-02 ⱡ | 1.30 |  |
| Asparagine | C_4_H_8_N_2_O_3_ | 116.0883 | 14.95 | - | - | 5.35 | GC-QTOF-MS | - | 1 | 0.97 | - | 1.51 |  |
| Glutamine | C_5_H_10_N_2_O_3_ | 156.0839 | 16.07 | - | - | 9.07 | GC-QTOF-MS | - | 1 | 0.87 | - | 1.48 |  |
| Serine | C_3_H7NO3 | 204.1226 | 11.13 | - | - | 6.39 | GC-QTOF-MS | - | 1 | 0.76 | 1.17E-02 | - |  |
| Tryptophan | C_11_H_12_N_2_O_2_ | 202.1047 | 20.41 | - | - | 22.57 | GC-QTOF-MS | - | 1 | 0.51 | 1.19E-02 | 2.03 |  |
| Methyl proline | C_6_H_11_NO_2_ | 84.0803 | 8.34 | - | - | 10.22 | GC-QTOF-MS | - | 1 | 2.16 | - | 1.14 |  |
| ***Carnitines*** | | | | | | | | | | | | |  |
| Carnitine | C_7_H_15_NO_3_ | 161.1052 | 1.08 | 5 | [M+H]^+^ | 3.50 | GM-RP-LC-QTOF-MS | ESI + | 2 | 1.28 | 2.00E-02ⱡ | - |  |
| Acetylcarnitine | C_9_H_17_NO_4_ | 203.1158 | 1.11 | 4 | [M+H]^+^ | 5.40 | GM-RP-LC-QTOF-MS | ESI + | 2 | 1.29 | 2.58E-02 | - |  |
| Propionylcarnitine | C_10_H_19_NO_4_ | 217.1314 | 1.14 | 3 | [M+H]^+^ | 3.08 | GM-RP-LC-QTOF-MS | ESI + | 2 | 1.88 | - | 1.14 |  |
| Octanoylcarnitine | C_15_H_29_NO_4_ | 287.2097 | 6.71 | 4 | [M+H]^+^ | 5.03 | GM-RP-LC-QTOF-MS | ESI + | 2 | 0.56 | 2.15E-02 | - |  |
| Arachidonoyl histidine | C_26_H_39_N_3_O_3_ | 441.2991 | 10.29 | 9 | [M+Na]^+^ | 4.35 | GM-RP-LC-QTOF-MS | ESI + | 3 | 5.09 | 3.08E-02 | 1.55 |  |
| Decanoylcarnitine | C_17_H_33_NO_4_ | 315.2410 | 11.33 | 5 | [M+H]^+^ | 2.07 | GM-RP-LC-QTOF-MS | ESI + | 2 | 0.57 | 1.93E-02 | - |  |
| Palmitoleoylcarnitine | C_23_H_43_NO_4_ | 397.3192 | 20.62 | 6 | [M+H]^+^ | 8.01 | GM-RP-LC-QTOF-MS | ESI + | 3 | 1.41 | 3.10E-02 | - |  |
| ***Indoles*** | | | | | | | | | | | | |  |
| Tryptophanol | C_10_H_11_NO | 161.0841 | 21.71 | 3 | [M+Na]^+^ | 2.23 | GM-RP-LC-QTOF-MS | ESI + | 3 | 0.78 | 1.22E-02 | 1.38 |  |
| ***Bile acids*** | | | | | | | | | | | | |  |
| Glycochenodeoxycholic acid sulfate | C_26_H_43_NO_8_S | 529.2709 | 9.44 | 2 | [M-H]^-^ | 4.55 | GM-RP-LC-QTOF-MS | ESI - | 3 | 9.82 | 1.97E-03ⱡ | 1.98 |  |
| Glycocholic Acid | C_26_H_43_NO_6_ | 465.3090 | 10.15 | 3 | [M-H]^-^ | 5.26 | GM-RP-LC-QTOF-MS | ESI - | 3 | 4.10 | 1.62E-03ⱡ | 1.98 |  |
| Taurochenodeoxycholic acid | C_26_H_45_NO_6_S | 499.2968 | 10.36 | 1 | [M-H]^-^ | 4.58 | GM-RP-LC-QTOF-MS | ESI - | 3 | 4.46 | 3.58E-03ⱡ | 1.77 |  |
| Deoxycholic acid 3-glucuronide | C_30_H_48_O_10_ | 568.3248 | 10.14 | 1 | [M-H]^-^ | 7.32 | GM-RP-LC-QTOF-MS | ESI - | 3 | 1.78 | 7.73E-03ⱡ | 1.04 |  |
| Glycodeoxycholic acid | C_26_H_43_NO_5_ | 449.3141 | 13.62 | 1 | [M-H]^-^ | 4.38 | GM-RP-LC-QTOF-MS | ESI - | 3 | 2.09 | 2.54E-02ⱡ | 1.33 |  |
| Hyodeoxycholic acid | C_24_H_40_O_4_ | 392.2927 | 18.36 | 2 | [M-H]^-^ | 4.52 | GM-RP-LC-QTOF-MS | ESI - | 3 | 0.45 | 1.06E-02ⱡ | 1.60 |  |
| Dehydroteasterone | C_28_H_46_O_4_ | 446.3396 | 34.33 | 2 | [M-H]^-^ | 10.72 | GM-RP-LC-QTOF-MS | ESI - | 3 | 0.60 | 6.50E-03ⱡ | 1.30 |  |
| 3,7,12-Trioxochola-1,4-dien-24-oic Acid | C_24_H_30_O_5_ | 398.2093 | 9.01 | 9 | [M-H]^-^ | 4.58 | GM-RP-LC-QTOF-MS | ESI - | 3 | 0.51 | 3.38E-03ⱡ | 1.69 |  |
| 1,3-Dihydroxy-5-cholan-24-oic Acid | C_24_H_40_O_4_ | 392.2927 | 15.08 | 0 | [M-H]^-^ | 8.48 | GM-RP-LC-QTOF-MS | ESI - | 3 | 0.19 | 9.28E-03ⱡ | - |  |
| ***Imidazopyrimidines*** | | | | | | | | | | | | |  |
| Uric acid | C_5_H_4_N_4_O_3_ | 168.0283 | 19.43 | - | - | 29.09 | GC-QTOF-MS | - | 1 | 0.36 | 4.56E-02ⱡ | 2.19 |  |
| ***Organic acids*** | | | | | | | | | | | | |  |
| Pyruvic acid | C_3_H_4_O_3_ | 88.0160 | 6.60 | - | - | 5.28 | GC-QTOF-MS | - | 1 | 3.23 | 2.20E-04ⱡ | 2.05 |  |
| Citric acid | C_6_H_8_O_7_ | 192.0270 | 16.64 | - | - | 2.67 | GC-QTOF-MS | - | 1 | 0.42 | 3.53E-04ⱡ | 1.81 |  |
| Glycolic acid | C_2_H_4_O_3_ | 76.0160 | 6.99 | - | - | 6.81 | GC-QTOF-MS | - | 1 | 0.76 | 1.79E-02ⱡ | - |  |
| Hydroxyglutaric acid | C_5_H_8_O_5_ | 148.0372 | 13.82 | - | - | 6.55 | GC-QTOF-MS | - | 1 | 10.72 | 2.08E-02 | 1.50 |  |
| Urea | CH_4_N_2_O | 60.0324 | 9.36 | - | - | 3.35 | GC-QTOF-MS | - | 1 | 0.81 | 1.35E-02ⱡ | - |  |
| ***Organooxygen compounds*** | | | | | | | | | | | | |  |
| Arabinose | C_5_H_8_O_5_ | 150.0528 | 14.99 | - | - | 8.80 | GC-QTOF-MS | - | 1 | 1.47 | 4.12E-02ⱡ | - |  |
| Ribose | C_5_H_8_O_5_ | 150.0528 | 15.14 | - | - | 7.45 | GC-QTOF-MS | - | 1 | 1.85 | 4.96E-03ⱡ | 1.32 |  |
| Gluconic acid | C_6_H_12_O_7_ | 196.0583 | 17.47 | - | - | 6.89 | GC-QTOF-MS | - | 1 | 0.57 | - | 1.08 |  |
| Isopropyl thiogalactopyranoside | C_9_H_18_O_5_S | 238.0875 | 12.62 | - | - | 12.62 | GC-QTOF-MS | - | 1 | 1.31 | 4.26E-02ⱡ | - |  |
| Galactose | C_6_H_12_O_6_ | 180.0634 | 3.00 | - | - | 3.00 | GC-QTOF-MS | - | 1 | 1.16 | 4.14E-02 | - |  |
| Allose | C_6_H_12_O_6_ | 180.0634 | 5.51 | - | - | 5.51 | GC-QTOF-MS | - | 1 | 1.29 | 1.35E-02ⱡ | - |  |
| Myo-Inositol | C_6_H_12_O_6_ | 180.0634 | 19.43 | - | - | 9.12 | GC-QTOF-MS | - | 1 | 1.81 | 2.79E-02ⱡ | 1.17 |  |
| ***Phenols*** | | | | | | | | | | | | |  |
| Cresol | C_7_H_8_O | 165.0722 | 8.10 | - | - | 4.07 | GC-QTOF-MS | - | 1 | 0.72 | 1.35E-02ⱡ | 1.61 |  |
| ***Pyrimidine nucleosides*** | | | | | | | | | | | | |  |
| Methyluridine | C_10_H_14_N_2_O_6_ | 258.0852 | 22.38 | - | - | 18.64 | GC-QTOF-MS | - | 1 | 0.82 | - | 1.30 |  |
| Rt: retention time; ^a^CV: coefficient of variation in the metabolites in the QC samples; ^b^Identification level: level 1 structure confirmed, level 2 structure probable, level 3 unequivocal molecular formula (s), level 4 exact mass; ^c^Change: change in the abundance of the specified comparison calculated as (case/control); ^d^*p* value ⱡ: corresponding to the *p* values calculated by the Benjamini-Hochberg false discovery rate post hoc correction (FDR < 0.05); ^e^VIP: variable importance in projection; GM: global metabolomics; LC: liquid chromatography; GC: gas chromatography; QTOF-MS: quadrupole time-of-flight mass spectrometer; LPC: lysophosphatidylcholines; PC: phosphatidylcholines; LPE: lysophosphatidylethanolamine; PE: phosphatidylethanolamine; SM: sphingomyelin; Cer: ceramides; MG: monoacylglycerol; DG: diacylglycerol; TG: triacylglycerol; HexCer: hexosylceramide; PG: phosphatidylglycerol; PI: phosphatidylinositol; PA: phosphatidic acid; LPA: lysophosphatidic acid; PS: phosphatidylserine; CDP-DG: cytidine diphosphate diacylglycerol; CE: cholesterol ester. | | | | | | | | | | | | |  |
|  |  |  |  |  |  |  |  |  |  |  |  |  |  |
|  |  |  |  |  |  |  |  |  |  |  |  |  |  |
|  |  |  |  |  |  |  |  |  |  |  |  |  |  |

| **SUPPLEMENTARY TABLE 3. TAC CONCENTRATION EXPRESSED IN TEAC μM BY GROUP** | | | |
| --- | --- | --- | --- |
| **GROUP** | **Q1** | **Q2** | **QT** |
| **AL Patients** | 80 ± 35.7 | 192 ± 40.1 | 314 ± 67.1 |
| **Healthy Individuals** | 130 ± 38.4 | 202 ± 55.4 | 375 ± 91.4 |
| TAC: Total Antioxidant Capacity; AML: Acute myeloid leukemia; B-ALL: B-acute lymphoid leukemia; Q1: Fast antioxidants; Q2: Slow antioxidants; QT: Q1+Q2. Mean±SEM | | | |

1. **Supplemental Figures**


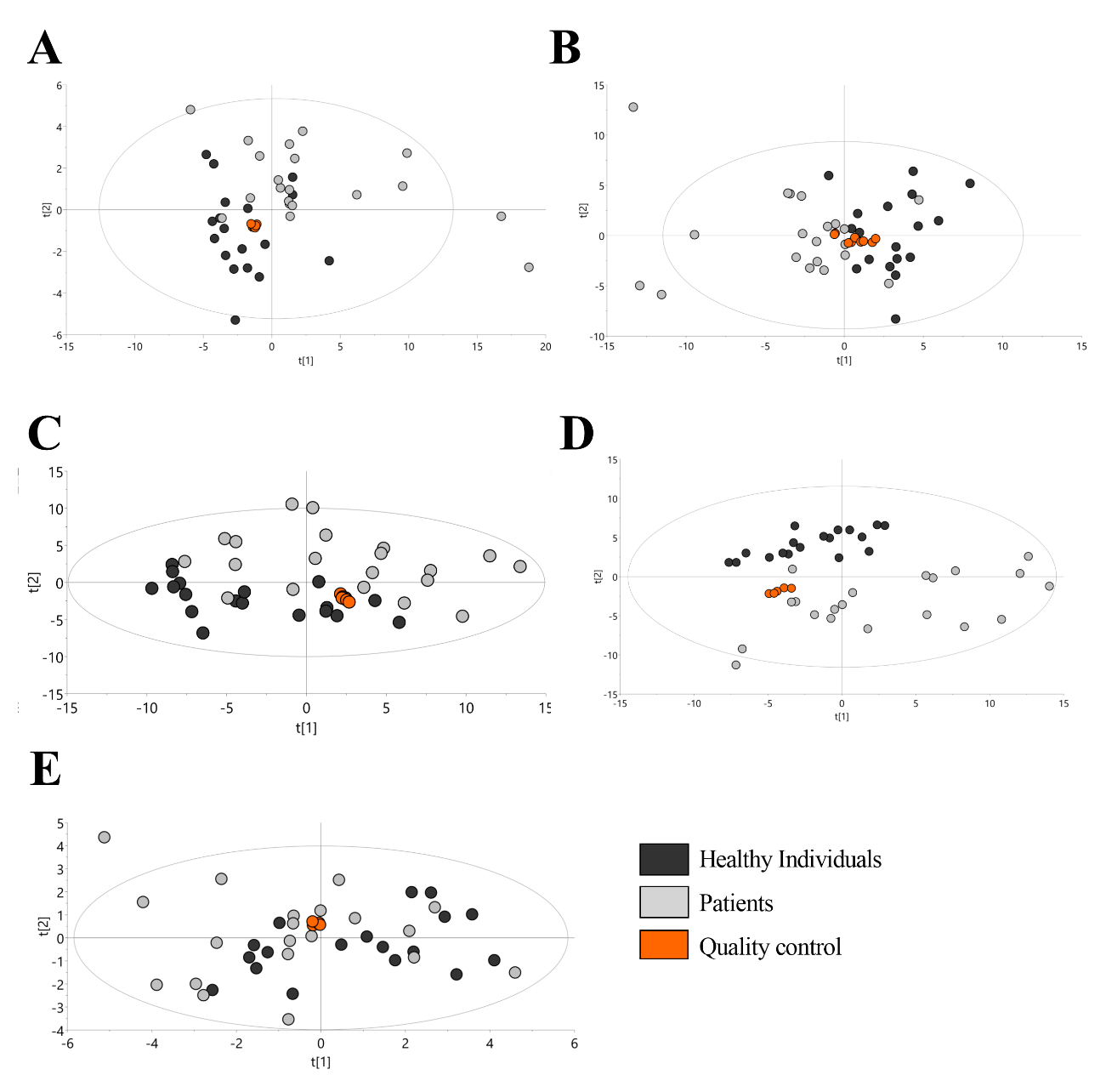


**SUPPLEMENTARY FIGURE 1 PCA score plots.** PCA score plots for data set filtered by presence and reproducibility. A. GM-LC/MS (+) R^2^: 0.604, Q^2^: 0.353. B. GM-LC/MS (-) R^2^: 0.752, Q^2^: 0.540. C. GL-LC/MS (+) R^2^: 0.818, Q^2^: 0.643. D. GL-LC/MS (-) R^2^: 0.746, Q^2^: 0.571. E. GC/MS (+) R^2^: 0.716, Q^2^: 0.0529. Dots in orange color denote quality control and gray dots correspond to samples (Healthy individuals and AL patients).


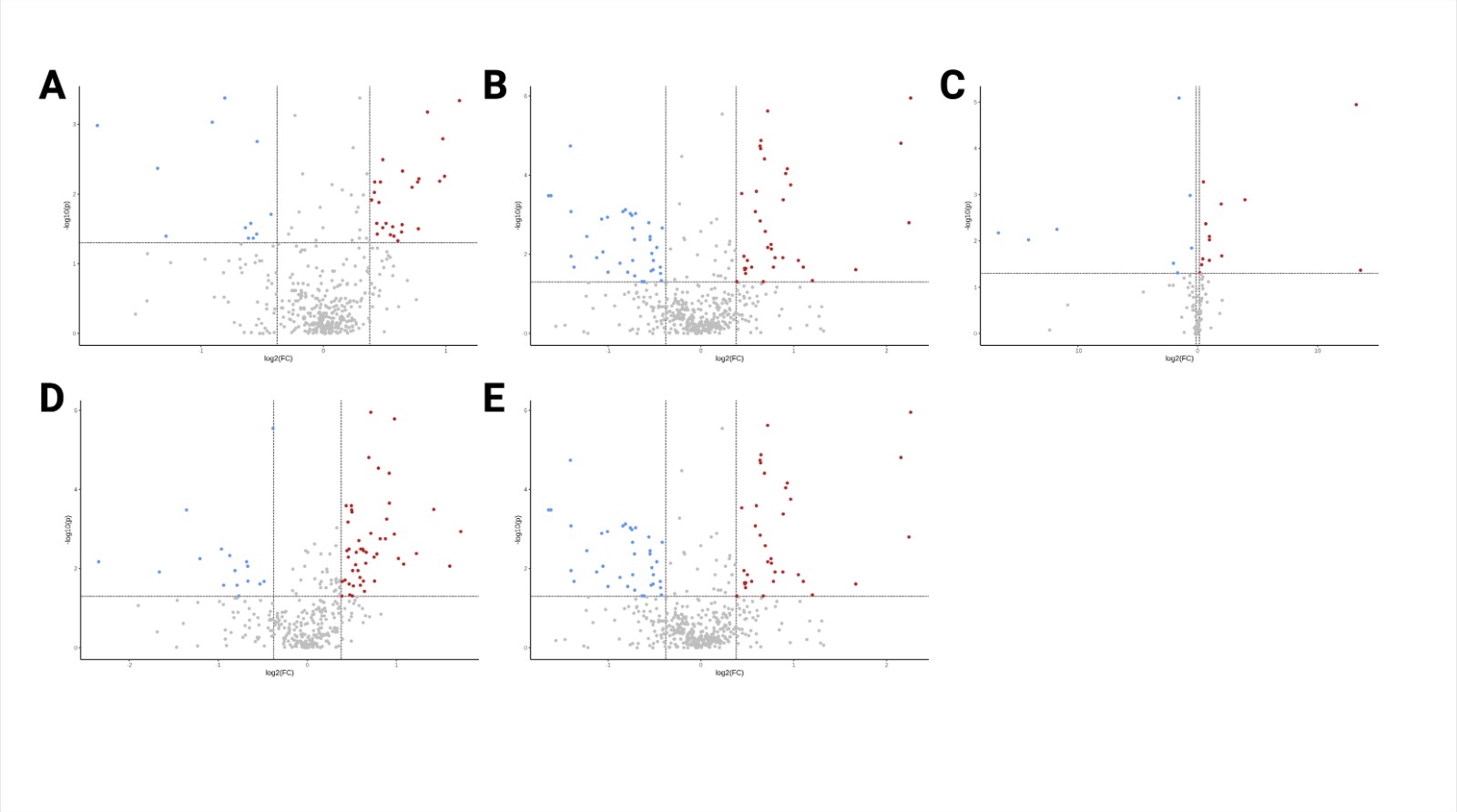


**SUPPLEMENTARY FIGURE 2 Volcano plots.** A. GM-LC/MS (+). B. GL-LC/MS (+). C. GC/MS (+). D. GM-LC/MS (-). E. GL/MS (-). Dots in blue color denote decrease features and red dots correspond to increased features.


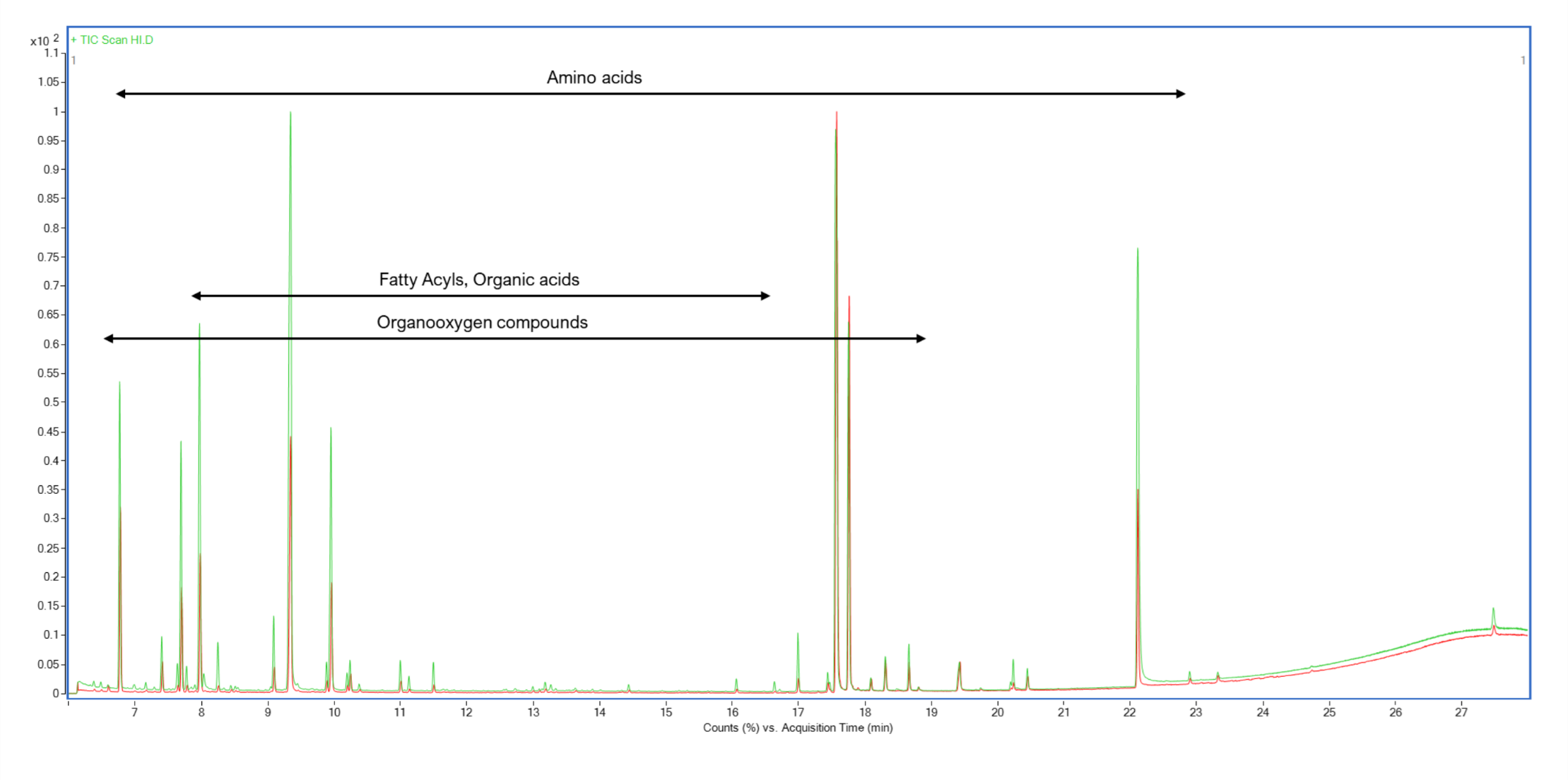


**SUPPLEMENTARY FIGURE 3 Comparison of GC-MS base peak chromatograms.** Comparison of GC-MS base peak chromatograms for AL patients (red) and healthy individuals (green).


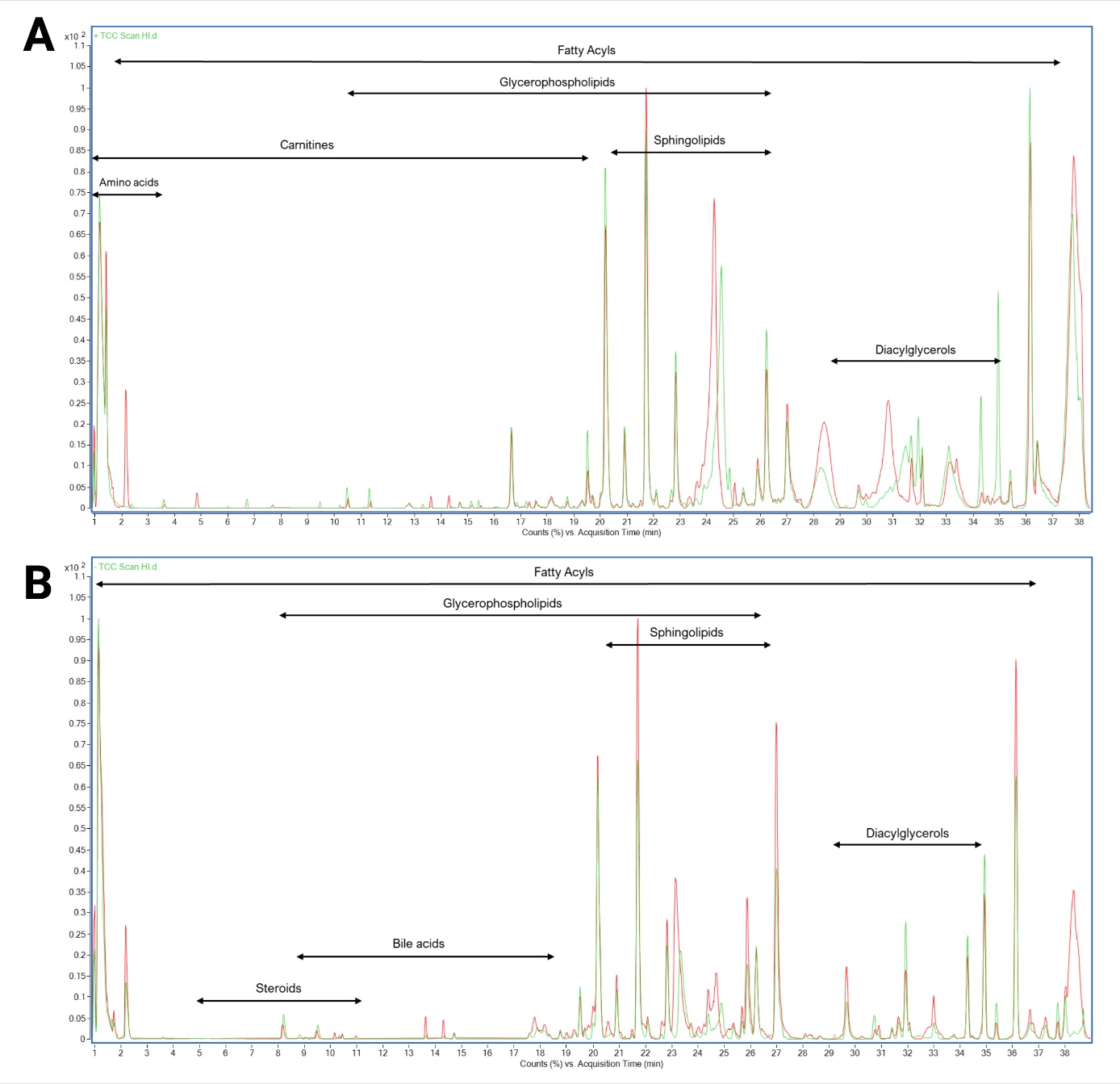


**SUPPLEMENTARY FIGURE 4 Comparison of GM-LC/MS base peak chromatograms**. Comparison of GM-LC/MS base peak chromatograms for AL patients (red) and Healthy individuals (green). Panel A. Metabolic fingerprinting by GM-LC/MS (+). B. Metabolic fingerprinting by GM-LC/MS (-).


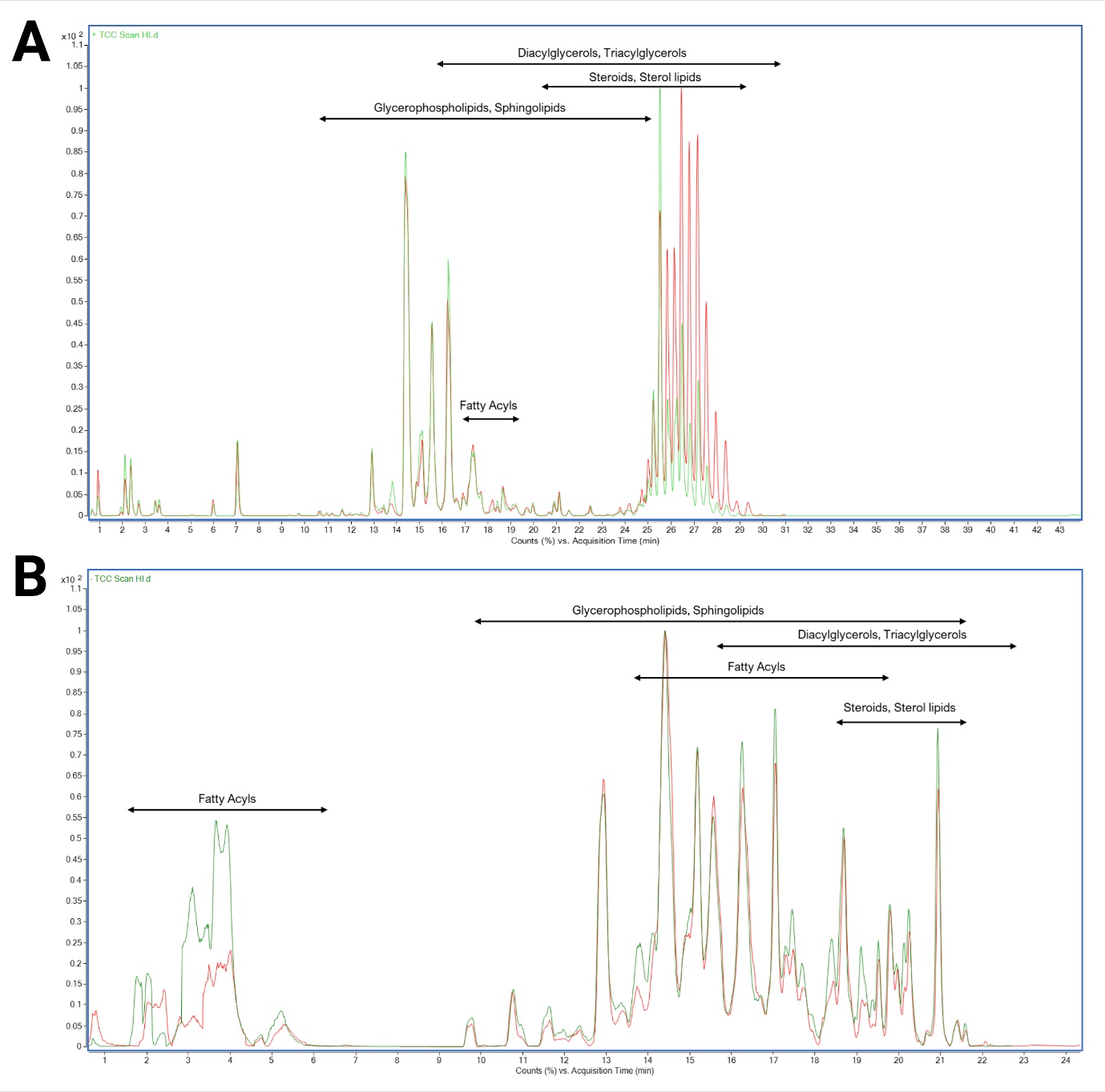


**SUPPLEMENTARY FIGURE 5 Comparison of GL-LC/MS base peak chromatograms**. Comparison of GL-LC/MS base peak chromatograms for patients (red) and Healthy individuals (green). Panel A. Lipid fingerprinting by GL-LC/MS (+). B. Lipid fingerprinting by GL-LC/MS (-).
